# Effectiveness of an Intervention to Improve HIV Service Delivery for People Who Inject Drugs in Kazakhstan: A Cluster Trial

**DOI:** 10.1001/jamanetworkopen.2022.44734

**Published:** 2022-12-01

**Authors:** Nabila El-Bassel, Tara McCrimmon, Elwin Wu, Mingway Chang, Assel Terlikbayeva, Timothy Hunt, Meruyert Darisheva, Sholpan Primbetova, Alissa Davis, Lisa R. Metsch, Daniel J. Feaster, Baurzhan Baiserkin, Asylkhan Abishev, Alfiya Denebayeva, Beibit Sagimbayev, Kulpan Kurmetova, Kozhakhmet Mashirov, Louisa Gilbert

**Affiliations:** 1 Social Intervention Group, Global Health Research Center of Central Asia, Columbia University School of Social Work, New York, New York; 2 Columbia University Mailman School of Public Health, New York, New York; 3 Global Health Research Center of Central Asia, Almaty, Kazakhstan; 4 Department of Public Health Sciences, Leonard M. Miller School of Medicine, University of Miami, Miami, Florida; 5 Medical and Pharmaceutical Control Committee of the Ministry of Health of the Republic of Kazakhstan, Almaty, Kazakhstan; 6 Kazakh Scientific Center of Dermatology and Infectious Diseases, Almaty, Kazakhstan; 7 Center for Prevention and Control of AIDS of the Almaty Health Department, Almaty, Kazakhstan; 8 Karaganda Oblast Center for Prevention and Control of AIDS of the Health Department of Karaganda Oblast, Karaganda, Kazakhstan; 9 Center for Prevention and Control of AIDS of the Shymkent Health Department, Shymkent, Kazakhstan

## Abstract

**Importance:**

The increasing HIV incidence rates and suboptimal rates of testing, engagement, and retention in care for people who inject drugs (PWID) in Kazakhstan underscore the need for effective HIV care continuum interventions for PWID.

**Objective:**

To determine the effectiveness of the Bridge HIV care continuum intervention implemented in needle and syringe programs (NSPs) in Kazakhstan.

**Design, Setting, and Participants:**

This stepped-wedge cluster trial was conducted from February 2017 to May 2020, with implementation beginning sequentially across 3 cities (Almaty, Karaganda-Temirtau, and Shymkent) in August 2017, January 2018, and May 2019. Intervention effect sizes were estimated via population-averaged models, and hypothesis testing relied on a permutation testing approach. The primary unit of analysis was an NSP. Data analysis was performed from October 2020 to April 2022.

**Interventions:**

The intervention addresses the full HIV care continuum: identification, testing, referral to services, and linkage to HIV care. The 3 intervention components were (1) a social network strategy, a peer-driven recruitment approach for HIV testing; (2) HIV counseling, rapid testing, and referral following international and national guidelines and protocols; and (3) enhanced antiretroviral treatment and access to services.

**Main Outcomes and Measures:**

The primary outcomes were the effectiveness of implementing Bridge’s enhanced service integration approach in increasing the number of PWID served at NSPs, increasing the number of PWID who are tested for HIV in NSPs, and improving linking HIV-positive PWID with HIV care. Secondary outcomes included numbers of clients registered for HIV care, initiation of antiretroviral therapy, and viral suppression.

**Results:**

Twenty-four NSPs (8 in each city) served a total of 1225 PWID (369 in Almaty, 618 in Karaganda-Temirtau, and 238 in Shymkent) at the preimplementation study step; 1015 clients (82.9%) were male, and the mean (SD) age was 36.7 (7.1) years. Compared with preimplementation study steps, during Bridge intervention implementation steps, NSPs experienced a significant increase in the number of PWID clients registered (incidence rate ratio, 2.37; 95% CI, 1.48-3.78) and the number of PWID who received rapid HIV tests (incidence rate ratio, 3.98; 95% CI, 2.30-6.90). No significant increase in referral to HIV care was observed. The study also found significant support for secondary outcomes of antiretroviral therapy initiation and the number of clients who achieved viral suppression.

**Conclusions and Relevance:**

In this stepped-wedge cluster trial, the findings suggest that implementation of the Bridge intervention was associated with significant improvement in several steps in the continuum of HIV care for PWID in Kazakhstan.

**Trial Registration:**

ClinicalTrials.gov Identifier: NCT02796027

## Key Points

**Question:**

Is a 3-component intervention (Bridge) that promotes client recruitment, HIV testing, and linkage to HIV treatment associated with improved engagement along the HIV care continuum among people who inject drugs (PWID) served by needle and syringe programs (NSPs) in Kazakhstan?

**Findings:**

A stepped-wedge cluster trial of 1225 PWID served by 24 NSPs demonstrated that implementation of the Bridge intervention was associated with significant increases in the number of PWID clients served (by 2.37 times) and tested for HIV (by 3.98 times) over a 4-year study period from 2017 to 2020. The Bridge intervention was not associated with a significant increase in the number of clients referred to HIV care from NSPs.

**Meaning:**

These findings suggest that an integrated intervention that addresses multiple components of the HIV care continuum shows potential to improve engagement and retention in HIV testing, treatment, and care for PWID served by NSPs in Kazakhstan.

## Teaser

This cluster trial examines the effectiveness of the Bridge HIV care continuum intervention implemented in needle and syringe programs for people who inject drugs in Kazakhstan.

## Introduction

Kazakhstan’s HIV epidemic has grown exponentially, from 1900 new cases in 2010 to 3300 new cases in 2020, representing a 73% increase in HIV incidence.^1^ Injection drug use remains a major mode of transmission, accounting for 20% of new HIV cases in 2020.^2^ Biannual surveillance has estimated that people who inject drugs (PWID) have the highest prevalence of HIV (8.3%) compared with other key populations in Kazakhstan (1.4% for sex workers, 6.5% for men who have sex with men, and 4.1% for incarcerated individuals).^3^ Furthermore, PWID have poor outcomes across the HIV care continuum. According to the Kazakh Scientific Center for Dermatology and Infectious Disease (the Ministry of Health division overseeing Kazakhstan’s HIV response), fewer than 80% of PWID know their HIV status.^2^ In 2020, 71% of known HIV-positive PWID received antiretroviral treatment (ART), and 85% of them had a viral load below Kazakhstan’s threshold for viral suppression (1000 copies/mL).^2^ Untreated HIV-positive PWID are at elevated risk for comorbidity and mortality, as well as HIV transmission through sexual and injection drug use networks.^4^

An array of multilevel factors contribute to suboptimal progress in the HIV care continuum for PWID.^5,6,7,8,9^ PWID face economic and social marginalization and pervasive stigma from communities and medical professionals. Kazakhstan’s harsh drug policies coupled with criminalization of drug use and discrimination also prevent PWID from accessing testing and treatment services.^10,11,12^ Finally, the fragmented nature of services in Kazakhstan makes it difficult for PWID to engage in the continuum of care.^5^

Differentiated service delivery is a client-centered approach that promotes the relocation of HIV testing, care, and treatment services from traditional medical facilities into local peer networks and community-based organizations.^13^ Kazakhstan’s network of 137 needle and syringe programs (NSPs) is an untapped venue to deliver HIV prevention and treatment services. NSPs are staffed by nurses, social workers, and outreach workers (often current or former PWID with strong community links) and are widely accessible through locations at AIDS centers (HIV care and treatment centers), primary health care clinics (known as polyclinics), and nongovernmental organizations (NGOs). They distribute condoms and syringes and conduct rapid HIV testing and referrals to local AIDS centers for confirmatory testing, case registration, and treatment. Approximately one-half (47.5%) of Kazakhstan’s estimated 94 600 PWID attend NSPs,^2^ but services have not been well integrated with AIDS centers, nor have they actively promoted HIV treatment. NSP staff have limited training in evidence-based approaches for recruitment or counseling. Research from other settings has shown the effectiveness of integrating HIV treatment into harm reduction settings.^14,15,16,17,18^ However, prior interventions have focused on only 1 aspect of HIV care for PWID (ie, rapid testing). Evidence-based interventions are needed to strengthen the capacity of NSPs to engage PWID along multiple steps of the HIV care continuum.

Our study builds on existing literature regarding differentiated service delivery approaches to HIV service delivery.^14,15,16,19^ We conducted a stepped-wedge cluster trial of the Bridge intervention at 24 NSPs in Kazakhstan from 2017 to 2020. This trial collected organization-level data on the effectiveness of delivering a 3-component integrated intervention using 4 implementation strategies. We hypothesized that during the Bridge intervention, compared with time periods before implementation (akin to standard practice), we would observe improvements in primary outcomes, including increased numbers of PWID served at NSPs, increased numbers of HIV rapid testing among PWID, and increased numbers of referrals to HIV care among PWID who test positive. We also hypothesized that during the Bridge intervention, compared with prior time periods, we would see improvements in secondary outcomes, including increased numbers of PWID registered for HIV care, increased numbers of PWID initiating of antiretroviral treatment, and increased numbers of PWID who achieve viral suppression.

## Methods

Although the complete Bridge study is a hybrid type II trial testing both effectiveness and implementation outcomes,^20^ this article focuses on the effectiveness primary outcomes; study protocols are shown in Supplement 1. All research activities involving human participants received approvals from the institutional research board at Columbia University and the ethics committee of the Kazakhstan School of Public Health. This study follows Consolidated Standards of Reporting Trials (CONSORT) reporting guidelines for stepped-wedge trials.

### Stepped-Wedge Design

**Figure 1.**
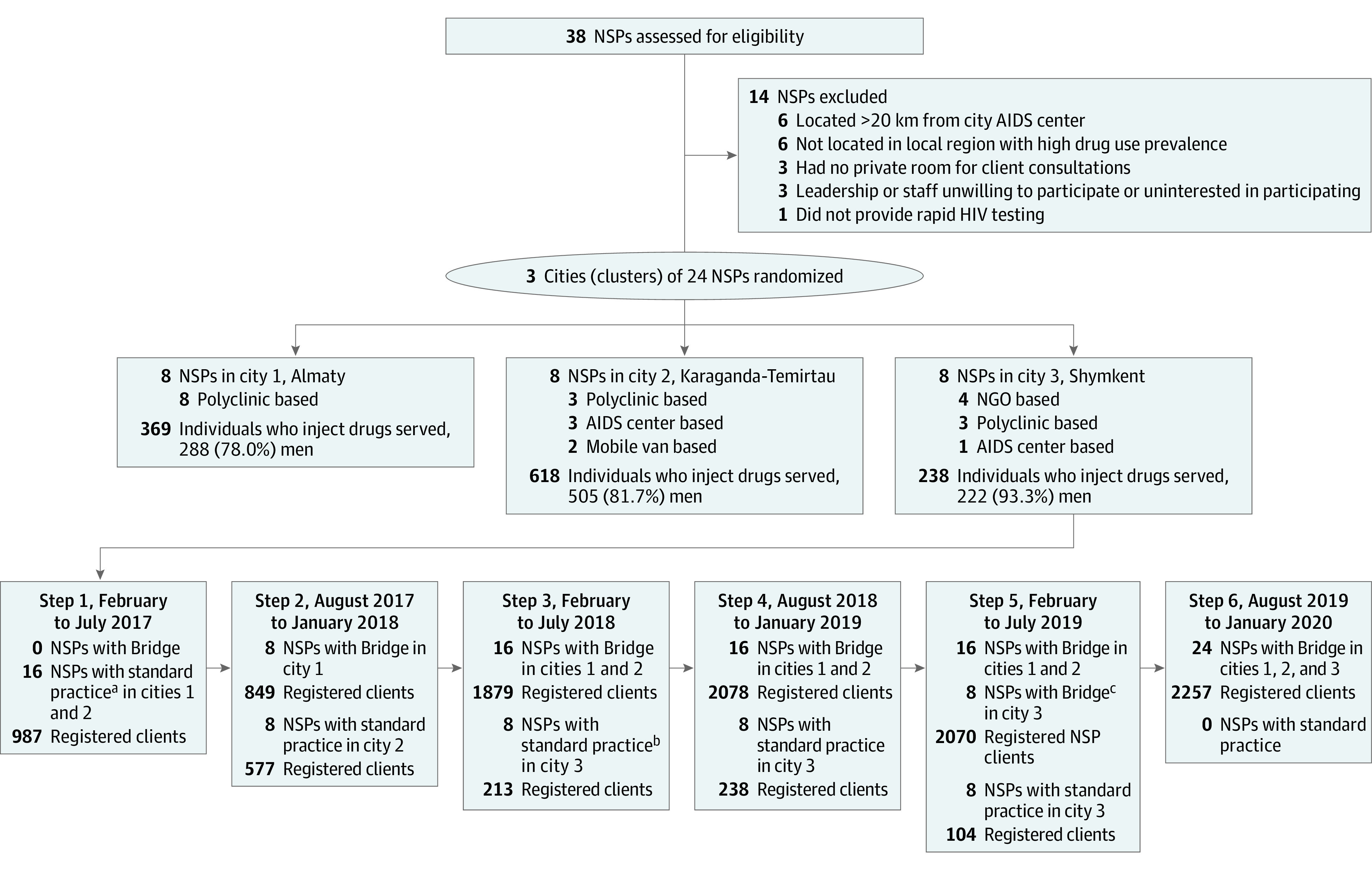
Diagram of Trial Participation NGO indicates nongovernmental organization; NSP, needle and syringe program. ^a^Refers to standard practice preimplementation of Bridge intervention. ^b^Because of organizational turnover, city 3 began preintervention standard practice in May 2018. ^c^After 12 months of preintervention standard practice, city 3 began Bridge in May 2019.

The study used a stepped-wedge cluster trial design in 3 cities (24 NSPs in total, 8 per city) (Figure 1). As an alternative to a randomized clinical trial, the stepped-wedge design ensures that all study sites will eventually receive the intervention, balancing important ethical and scientific considerations.^21^ The study was conducted from February 2017 to March 2020. Study steps were 6 months each in duration, with step 1 beginning in February 2017, step 2 beginning in August 2017, and so forth. A total of six 6-month study steps were used across all 3 cities (Figure 1). After 6 months of standard care preimplementation data collection (step 1), the intervention was initiated in city 1 (Almaty) at the start of step 2, followed by city 2 (Karaganda-Temirtau) at the start of step 3. Implementation for city 3 (Shymkent) was scheduled to begin with step 4. However, after experiencing closures of 4 NGO-based NSPs and staff redeployment during step 3, we delayed the start of preimplementation data collection until May 2018 (during step 5) and implemented the intervention in May 2019.

### Site Selection for Stepped-Wedge

We selected the cities of Almaty, Shymkent, and Karaganda-Temirtau (2 cities considered as 1, given their geographic proximity and shared administrative oversight) for their high prevalence of injection drug use, and enough NSPs and estimated numbers of HIV-positive PWID who remained unlinked to care. Detailed selection criteria have been published elsewhere.^20^ We also considered the diversity of these cities in terms of population size, geographic locations, and racial and ethnic composition to enhance generalizability of study findings across Kazakhstan. We selected 8 NSPs as sites within each city that met the following criteria: (1) located within 20 km of the AIDS center; (2) provided rapid HIV testing as part of regular services; (3) had a private room available for confidential consultations and pretest and posttest HIV counseling; (4) located within regions that had highest numbers of PWID (as estimated by local AIDS centers); and (5) leadership and staff expressed willingness to take part in the study. NSP staff, AIDS center staff, and leadership and other stakeholders were not blinded to intervention assignment.

### Preimplementation Standard Practice

Standard NSP services in each city included HIV rapid test with finger prick and referrals to confirmatory testing and treatment services at AIDS centers. Services are provided free of charge to citizens of Kazakhstan in their region of residence. Standard recruitment at NSPs is conducted by outreach workers, and referrals do not entail case management or supports. Although NSP government protocols include procedures for testing and referrals, these practices are often implemented inconsistently across different NSPs.

### Bridge Intervention

The Bridge intervention integrates 3 evidence-based interventions into a single program that includes identification, testing, and linkage to care. The first component is a social network strategy,^22^ a peer-driven recruitment approach for HIV testing based on social network theory, with demonstrated effectiveness in reaching hidden populations. Two outreach workers per NSP identified and trained peer recruiters drawn from PWID community networks and provided coaching and support for sharing information about HIV testing at NSPs. Peer recruiters received a $2 incentive for each unique referral who completed HIV testing. The second is HIV counseling, rapid testing, and referral, conducted by NSP nurses and social workers in accordance with international (World Health Organization and Centers for Disease Control and Prevention) guidelines, as well as national protocols.^23,24^ The third is enhanced antiretroviral treatment and access to services (ARTAS),^25^ also conducted by nurses and social workers. This case management intervention supports recently diagnosed HIV-positive PWID as they link or relink (those who have been lost to follow-up) to HIV care and supports treatment adherence. Enhanced ARTAS took place at NSP locations in 5 or more sessions, depending on individual client needs and barriers, and included opportunities for linkage to other services such as drug treatment^26^ and tuberculosis-related and hepatitis-related care. Regional studies^27,28^ have also shown the effectiveness of ARTAS as a stand-alone intervention.

Staff at each NSP received intensive training to deliver the Bridge intervention in the month before implementation and ongoing supervision, technical support, and collective learning through a community of practice model. The research team regularly reviewed supervision records for quality assurance of intervention delivery. More details on these implementation strategies are available elsewhere.^20^ Nurses and social workers completed human participants protection training and conducted a brief verbal informed consent process with clients before enrollment.

### Outcome Measures and Data Collection

Descriptive measures of each of the 24 study NSPs (including location and administration of each NSP) were collected from study records. The outcome measures for each 6-month step, as well as NSP client characteristics at the first (full) preimplementation step, were collected through 2 assessment tools from NSPs or AIDS centers. Primary outcome measures were collected from NSPs. NSP visits were measured by the number of PWID who attended NSPs in each 6-month study step. HIV testing was measured by the number of PWID who received a rapid test for HIV at the NSP in each 6-month study step. Referral to care was measured by the number of PWID who were referred to HIV care at the AIDS center in each 6-month study step. These 3 measures were collected via point-of-care data entry at NSPs through a tablet-based program that created and updated an electronic case record for each client. Unique client identifications (IDs), unique IDs with recorded HIV testing, and unique client IDs with a positive HIV test and a record of an AIDS center referral were summed and averaged per month within each 6-month study step. Quality assurance measures included quarterly observations by field staff and regular monitoring of data by the study team. Further details on data collection procedures have been published elsewhere.^20^

Secondary outcome measures were collected from AIDS centers in each city. Linkage to care was measured by the number of HIV-positive PWID registered at the AIDS center. ART initiation was measured by the number of HIV-positive PWID who newly initiated ART at the AIDS center. Viral suppression was measured by the number of HIV-positive PWID who received ART and had a viral load level less than or equal to 1500 copies/mL. This cutoff was selected as an established indicator of a low viral load where HIV transmission is unlikely to occur.^29,30,31^ These measures were collected through patient records, which contained indicators unavailable through point-of-care data collection methods, such as laboratory test results. Medical record review used data from Kazakhstan’s National Electronic HIV Case Management System, a government-approved database for the collection, storage, transfer, and analysis of epidemiological, laboratory, and clinical data on all HIV-positive registered cases.^32^ Every 6 months, AIDS center staff extracted data from this repository on PWID clients who had attended the 24 study NSPs and entered them into a report format submitted to the researchers. Quality assurance measures included a check of a randomly selected 10% of AIDS center records during each 6-month reporting period.

### Statistical Analysis

Population-averaged models using a generalized estimating equation approach were used to assess the effectiveness of Bridge. The models used NSP as a unit of analysis and also account for repeated measures within NSPs. Population-averaged negative binominal regression was used to estimate intervention effects (as before vs after change); the estimate was reported as incident rate ratio (IRR) and 95% CIs. The population-averaged models for NSP visits, HIV testing, and referral to care included step number, to account for the variance due to measures over time; city, to account for the variance due to different locations; and the interaction terms between step number and cities. The population-averaged models for linkage to care, ART initiation, and viral suppression included city and the step number (without interaction terms) because only 1 NSP was recorded in AIDS center record review in Shymkent (city 3).

Because of concerns that the distribution of measures might not be approximated well because of the small sample size of 24 NSPs, we used permutation tests to determine statistical significance for hypothesis testing. Permutation tests could also guard against type I errors that might arise as a result of multiple comparisons.^33,34^ We permuted the Bridge implementation status of each step; since the step assignment (preimplementation standard care vs Bridge implementation) was allocated on the basis of city, permutations accounted for clustering by city. A total of 1000 random permutations were performed for each outcome. The observed intervention effect based on actual step assignments was compared vis-à-vis the distribution of the intervention effects calculated across all the permutations. The 2-sided *P* values were calculated as the proportion of absolute values for permuted effects greater than the absolute value of the actual observed effects. Formal hypothesis testing relied on the estimated *P* value from permutation tests, using a criterion of *P* < .05 (*P* < .025 for each side) to reject the null hypothesis. Stata statistical software version 15.1 (StataCorp) was used for all analyses. Data analysis was performed from October 2020 to April 2022.

## Results

**Figure 2.**
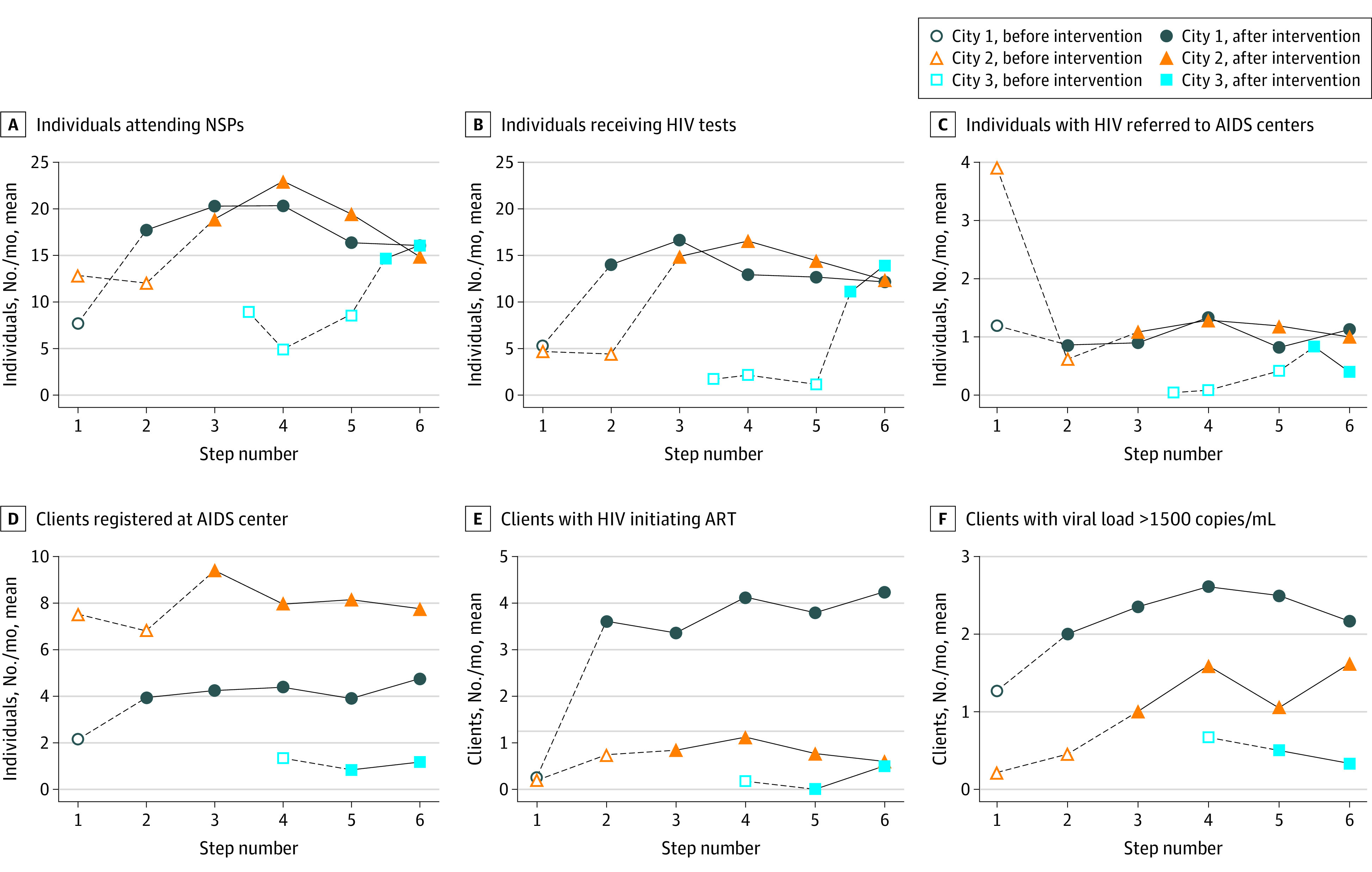
Outcome Measures Over Time ART indicates antiretroviral therapy; NSP, needle and syringe program.

Before implementation, the 24 study NSPs (8 in each city) served a total of 1225 unique PWID (369 in Almaty, 618 in Karaganda-Temirtau, and 238 in Shymkent). Most clients (1015 clients [82.9%]) were male, and the mean (SD) age was 36.7 (7.1) years. The overall study sample of 24 NSPs included 14 NSPs operated by government primary health care clinics, 6 operated by the regional AIDS center (including 2 mobile van–based NSPs), and 4 operated by NGOs. There was, however substantial variation in clients served, client sociodemographic characteristics, and NSP administration by city. The descriptive characteristics of NSPs and their clients by study city are presented in Table 1. The means of the outcome measures per month by 3 cities are shown in Figure 2.

**Table 1.** Organizational and Client Characteristics by City

Characteristic	Almaty (city 1)	Karaganda-Temirtau (city 2)	Shymkent (city 3)
Organizational administration type, No. of NSPs^a^			
Primary health care clinic (polyclinic)	8	3	3
AIDS center			
Stationary (clinic-based)	0	3	1
Mobile van	0	2	0
Nongovernmental organization	0	0	4
Client characteristics at the first (full) preimplementation step			
Unique clients served in the city, total No.	369	618	238
Clients served in NSPs within the city, mean (SD) [range], No.	46.1 (3.8) [28.0-63.0]	77.3 (27.6) [11.0-230.0]	29.7 (28.4) [16.0-100.0]
Male clients served in the city, No. (%)	288 (78.0)	505 (81.7)	222 (93.3)
Male clients served in NSPs within the city, range, %	52.9-95.1	62.5-86.4	78.9-100.0
Age of clients served in the city, mean (SD) [range], y	36.0 (7.7) [18.0-60.0]	35.9 (5.9) [22.0-59.0]	39.9 (7.8) [24.0-56.0]
Mean age of clients served in NSPs within the city, range, y	30.9-42.3	33.2-39.8	34.3-43.8

Abbreviation: NSP, needle and syringe programs.

^a^ There were 8 NSPs in each city.

Findings for the hypothesis tests of intervention effects (before vs after change) in NSP visits, HIV testing, and referral to care are shown in Table 2, including *P* values derived from permutation tests. Compared with the preimplementation steps, the steps after Bridge implementation had significantly more clients attending NSPs (IRR, 2.37; 95% CI, 1.48-3.78) and more clients receiving HIV tests (IRR, 3.98; 95% CI, 2.30-6.90). However, the results did not show a significant increase in the number of referrals of HIV-positive clients to AIDS centers after implementation, compared with the preimplementation steps.

**Table 2.** Intervention Effect on Primary Outcomes via Population-Averaged Models

Variable	IRR (95% CI)		
	PWID who attend NSP per month	PWID who receive HIV tests per month	PWID who are referred to AIDS center per month
Intervention effect (before vs after change)	2.37 (1.48-3.78)	3.98 (2.30-6.90)	0.92 (0.51-1.69)
Step number	0.98 (0.85-1.12)	0.91 (0.78-1.06)	1.01 (0.85-1.20)
City 2^a^	1.75 (0.66-4.66)	0.97 (0.36-2.60)	2.64 (0.94-7.38)
City 3^a^	1.02 (0.18-5.83)	0.14 (0.02-1.04)	0.002 (0.0001-0.04)
Step number × city 2^a^	0.90 (0.76-1.07)	1.03 (0.84-1.25)	0.82 (0.67-1.02)
Step number × city 3^a^	0.98 (0.72-1.32)	1.41 (0.98-2.03)	2.78 (1.62-4.78)
Estimated *P* value associated with intervention effect from permutation test	<.001	<.001	.65

Abbreviations: IRR, incidence rate ratio; NSP, needle and syringe program; PWID, people who inject drugs.

^a^ City 2 is Karaganda-Temirtau, and city 3 is Shymkent.

Table 3 shows the findings of population-averaged models for linkage to care, ART initiation, and viral suppression and the permutation tests for significance. After the Bridge intervention was implemented, there were significantly more clients who newly initiated ART (IRR, 2.53; 95% CI, 1.52-4.22) and more clients whose viral loads were less than 1500 copies/mL (IRR, 1.84; 95% CI, 1.08-3.11), compared with preintervention times. However, the estimated effect for the number of clients registered at an AIDS center was not statistically significant because the *P* values from the permutation tests were greater than .025 for the 2-sided tests.

**Table 3.** Intervention Effect on Secondary Outcomes via Population-Averaged Models

Variable	IRR (95% CI)		
	HIV-positive NSP clients who register at AIDS Center per month	HIV-positive NSP clients who initiate ART per month	HIV-positive NSP clients who have a viral load ≤1500 copies/mL per month
Intervention effect (before vs after change)	1.14 (0.96-1.36)	2.53 (1.52-4.22)	1.84 (1.08-3.11)
Step number	1.03 (0.99-1.08)	1.07 (0.94-1.20)	1.09 (0.96-1.25)
City 2^a^	2.10 (0.76-5.76)	0.23 (0.09-0.58)	0.49 (0.20-1.20)
City 3^a^	0.28 (0.03-2.48)	0.07 (0.01-0.74)	0.23 (0.03-1.77)
Estimated *P* value associated with intervention effect from permutation test	.15	<.001	.007

Abbreviations: ART, antiretroviral therapy; IRR, incidence rate ratio; NSP, needle and syringe program.

^a^ City 2 is Karaganda-Temirtau and city 3 is Shymkent.

## Discussion

The findings of this cluster trial support our study hypotheses for our primary outcomes of clients served and HIV testing at NSPs, as well as our secondary outcomes of new ART enrollment and clients receiving ART with viral loads 1500 copies/mL or less. However, statistical tests failed to reject the null hypothesis for referrals and linkage to care at AIDS centers. Our findings show that the Bridge intervention model of differentiated service delivery has the potential to improve outcomes along multiple steps of the HIV care continuum for PWID in Kazakhstan. There was no evidence of significant differences in any outcome between study sites, which is particularly notable given the heterogeneity of organizational structures comprising the 8 NSPs in each city. The findings suggest that NSPs of all types were able to reach more marginalized groups of PWID through the innovative social network strategy component and test and link them to HIV care using the enhanced ARTAS approach. The lack of statistically significant findings regarding referrals and linkage to care require more careful consideration, including mixed-methods research to understand what factors may be responsible for this outcome. Additional research is needed to examine how factors such as client gender may moderate the effectiveness of the Bridge intervention. To our knowledge, Bridge is the first study to examine the effectiveness of an integrated continuum of HIV care for PWID who attend NSPs in Kazakhstan. Although other regional studies^27,28^ have identified the effectiveness of ARTAS case management in promoting linkage to HIV treatment, this is not easily comparable to the integrated Bridge intervention.

### Limitations and Strengths

There are several limitations to our study. Data entry may have been subject to user errors and staff biases to show positive outcomes. We tried to minimize these effects through quality assurance procedures as described previously. Because of NGO closures in city 3 and resulting staff turnover, the preimplementation data collection was delayed in this city until May 2018 and the intervention did not start until May 2019. This delay did not allow us to conduct statistical comparisons across cities at each step. In addition, we cannot attribute all outcomes entirely to Bridge as there may have been other non–intervention-related effects, such as changes in government funding and resources for HIV care continuum services, including access to rapid HIV test kits, that contributed to the increases in HIV testing, linkage to treatment, and viral load outcomes across the regions during the study period. Despite these limitations, this clinical trial has several strengths, including the innovative Bridge intervention, a multifaceted data collection system, and conduct of the intervention directly within local harm reduction programs, which increases the clinical practice relevance of the intervention.

## Conclusions

The Bridge intervention’s promising outcomes underscore its potential to augment the role that NSPs play in Kazakhstan’s existing HIV care system, transforming them from mere harm reduction programs into a single-source support for the HIV care continuum, as well as linkage to other key services for PWID. The dissemination of the Bridge intervention, both to other NSPs in Kazakhstan and other countries in the region and globally, may lessen the gaps in the HIV care continuum for PWID.

## Notes

Supplement 1.
Trial Protocol and Statistical Analysis Plan

Supplement 2.
Data Sharing Statement
