# Supplementary material for: Effectiveness of an Intervention to Improve HIV Service Delivery for People Who Inject Drugs in Kazakhstan: A Cluster Trial
Source: JAMA Netw Open. 2022 Dec 1;5(12):e2244734. doi: 10.1001/jamanetworkopen.2022.44734 (PMC9716389; doi:10.1001/jamanetworkopen.2022.44734)
Supplement: Supplement 1. — Trial Protocol and Statistical Analysis Plan [file jamanetwopen-e2244734-s001.pdf]

Columbia University Human Subjects Study Standalone Protocol
IRB of Record: Columbia University Morningside
Protocol: AAAQ0952 (Y01M00 – Y08M00)
Initial approval: 8/10/2015 (Y01M00)
Full Protocols Approved: 9/15/2016 (Y02M00)
Expiration Date: 05/26/2023 (Y08M00)
Originating Department: SSW Glb Hth Rsch Ctr Cent Asia (5762102)
Title: BRIDGE: Linking People who Inject Drugs to HIV Testing and Care
Abbreviated Title: BRIDGE
Principal Investigator: Nabila El-Bassel

BRIDGE
Stand-Alone Protocol for Effectiveness Outcomes

1. STUDY DESCRIPTION

Background: HIV and injection drug use in Kazakhstan

An estimated 1% of Kazakhstani adults (N=122,850) inject drugs. In some cities along drug trafficking routes, the rate is as high as 14%, representing one of the highest injection drug use rates in the world. As the epidemics of heroin use and HIV spread across Central Asia, there is an urgent need to scale up low-threshold highly effective HIV services for people who in inject drugs (PWID) in needle & syringe programs that may be integrated with HIV care to curb HIV transmission. Cities selected for this study - Almaty, Karaganda, Temirtau, and Shymkent - are geographically disparate and HIV prevalence rates among PWID range from 7% to 21%. Total populations range from 145,000 to over 1.5 million, which will ensure greater generalizability of study findings.

Figure 1: HIV Care Cascade for PWID in Kazakhstan

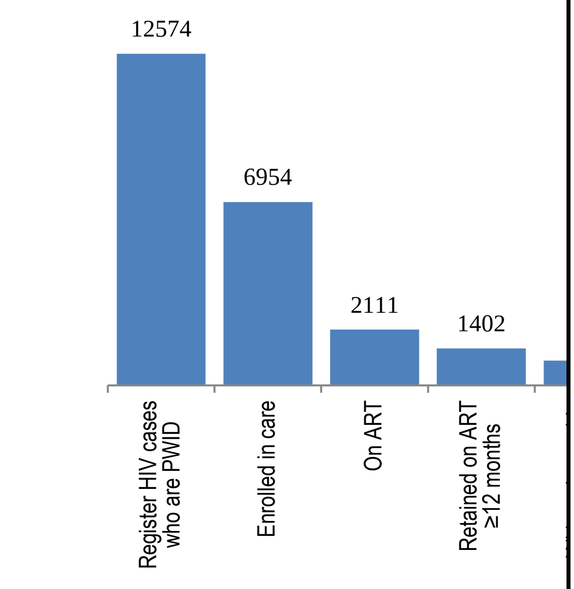

Sources: Republican AIDS Center 2014[27]

Major gaps in the HIV care continuum from HIV testing to virologic suppression among Kazakhstani PWID (see Figure 1) mirror gaps found among injection drug use driven HIV epidemics in Central Asia, Eastern Europe, and Asia. Only 4% (N=935) of the estimated 19,000 PWID living with HIV/AIDS in 7% of the registered 12,574 HIV-positive cases in Kazakhstan achieved viral suppression in 2014 as shown in Chart 1 This steep drop in the continuum of care among PWID is consistent with research worldwide among PWID HIV-positive PWID who remain untreated and viremic play a major role in increasing the epidemic in their communities Research suggests that, if offered the opportunity to be on ART, PWID are equally as likely to benefit from it as people living with HIV/AIDS who do not inject drugs ART initiation among PWID is associated with significant subsequent reductions in HIV incidence, unprotected sex and unsafe injections This research highlights the critical importance of identifying and linking or relinking PWID living with HIV to HIV care with an ultimate aim of initiating ART and virologic suppression. Currently 5,579 HIV-positive PWID with CD4 counts <500 will newly qualify for ART in Kazakhstan and need to be linked or relinked to HIV care to initiate ART.

Worldwide, needle & syringe programs (in Kazakhstan, more commonly known as Trust Points) have played a pivotal role in curbing the HIV epidemic by providing HIV prevention information along with syringes and condoms, and referring PWID to HIV testing and treatment. A recent meta-analyses of 12 studies with 12,000 person years found that Trust Points (TPs) significantly reduced the likelihood of HIV transmission with a pooled effect size of 0.66 TPs across Kazakhstan represent the largest single type of service provided to PWID dwarfing opiate substitution treatment (OST) and other drug treatment services. Kazakhstan contains over 140 TPs, all of which are located in "Trust Points", which also provide services to commercial sex workers, men who have sex with

men, and other high-risk groups. All trust points in Kazakhstan are regulated by the Republican AIDS Center. Estimates of TP coverage of PWID (at least one visit to a TP in the past year) in Kazakhstan range widely between 10%-47%. Mathematical modeling from a meta-analysis of TPs suggests that increasing coverage of TPs to 50% of PWID could reduce HIV incidence rates by 20% after 5 years and linking 50% of HIV-positive PWID with CD4 counts <500 to ART could result in a 20% decrease in the HIV incidence rate.

1.1. STUDY PURPOSE

The main aim of the proposed study is to increase linkages to care by evaluating the effectiveness of implementation, feasibility of expansion, and sustainability of an enhanced integrated HIV service package (BRIDGE) for Trust Points (TPs), also known as Needle Syringe Programs or Harm Reduction Programs for PWID) that includes low-threshold strategies of peer-driven recruitment, HIV counseling and testing (HCT), and ARTAS (CDC's recommended case management strategy to link PWID to HIV care) delivered by TP case managers who will be trained and supervised by TP nurses.

The BRIDGE intervention is systematically designed for PWID to address specific service barriers to HIV testing, and link them to HIV care and promote ART initiation. BRIDGE is an integrated intervention which combines the following three components: (1) Social Network Strategy (SNS), a peer-driven recruitment approach, which has demonstrated effectiveness in reaching hidden and hard to reach populations of unknown HIV status and successfully linking them to HIV testing; (2) HIV Counseling, Testing, and Referral (CTR), adhering to WHO and CDC guidelines for confidential, HIV Rapid Testing conducted by AIDS center nurses and accompanying confirmatory test; and (3) ARTAS, an evidence-based case-management intervention to link recently-diagnosed HIV-positive persons to care and retain them in care. The study builds on the investigative team's decade-long HIV intervention research with PWID in Kazakhstan carried out in collaboration with the Republican AIDS Center (RAC). It addresses implementation research questions to improve HIV service delivery systems for PWID that are not only important to the region, but have relevance for the 120 countries that have concurrent injection drug use and HIV epidemics. This study will employ an innovative design to evaluate implementation and effectiveness of BRIDGE on improving linkage to HIV care and initiation of ART in 24 TPs located in 4 geographically diverse Kazakhstani cities using a stepped wedge trial and site-level data collected from TPs and AIDS centers.

1.2. STUDY AIMS

Specific aims for the proposed research are:

Primary Aims

Aim 1: To evaluate the effectiveness of implementing BRIDGE's enhanced service integration approach in increasing number of PWID who are tested for HIV in TPs, and improving linking HIV-positive PWID to HIV care (primary outcomes).

Secondary Aims

Aim 2: To evaluate the effectiveness of BRIDGE's enhanced service integration approach on increasing retention in AIDS center, initiating ART, and increasing adherence to HIV treatment regimens and virologic suppression;

Aim 3: To assess how multi-level theory-driven factors (client, staff, agency, community, structural) influence the implementation and effectiveness of BRIDGE on Aim 1 and Aim 2 outcomes; and

Aim 4: To evaluate the implementation process and cost of BRIDGE including implications for cost-effectiveness, feasibility of expansion, and sustainability.

1.3. STUDY RATIONALE

Scaling up integrated, cost-efficient HIV services for people who inject drugs (PWID) in Needle Syringe Programs (TPs) is urgently needed in Kazakhstan, where only one-third of the estimated 19,000 HIV-positive PWID are ever linked to HIV care and only 10% initiate ART. The study's aim is to evaluate the implementation, effectiveness, and sustainability of an integrated HIV service model in 24 TPs in 4 cities that includes highly effective strategies of peer-driven recruitment of PWID using social network strategies (SNS), rapid HIV testing and counseling in TPs, and linkage of HIV positive PWID in TPs to HIV care using the ARTAS case management model. Findings will have important public health implications for improving HIV service delivery for PWID in the Central Asian region

and other countries with injection driven epidemics.

2. STUDY DESIGN & STATISTICAL PROCEDURES

The proposed study will evaluate the implementation and the effectiveness of the enhanced service integration approach of BRIDGE in trust points via a stepped wedge trial. A stepped wedge trial is a novel type of cluster trial design that is increasingly being used to evaluate service intervention. In a stepped wedge design there are random and sequential crossover of clusters from control to intervention until all clusters are exposed.

There are three main components to the study that are referred to throughout this protocol, each with their own sets of procedures and participants. The first is the BRIDGE intervention itself, described directly below and in the Intervention Manual included in this submission. As an implementation study, the BRIDGE team will train existing service providers to deliver this intervention to PWID at trust points in four study sites. Trust point staff will enroll PWID in each element of the intervention. We estimate that BRIDGE has the potential to reach up to 557 PWID per trust point (for a total of 13,381 PWID). Process measures, an electronic barcode-based tracking system known as AppSheet (described in Appendix A to this protocol), and medical records review referred to as the AIDS Center Semi-Annual Report (described in Appendix B to this protocol) will be used to assess outcomes from this study component.

2.1. OVERVIEW OF BRIDGE INTERVENTION

BRIDGE is an integrated intervention which combines the following three components: (1) Social Network Strategy (SNS), a peer-driven recruitment approach, guided by network theory which has demonstrated effectiveness in reaching hidden and hard to reach populations of unknown HIV status and successfully linking them to HIV testing as well as re-engaging HIV-positive PWID who never initiated or were never retained in care; (2) HIV Counseling, Testing, and Referral (CTR), adhering to WHO and CDC guidelines for confidential, HIV Rapid Testing conducted by AIDS center nurses and accompanying confirmatory test; and (3) ARTAS, a strengths-based case-management intervention to link recently-diagnosed HIV-positive persons to care and retain them in care. SNS, CTR, and ARTAS components have proven to be highly effective strategies used in many countries, including numerous studies by co-investigator Dr. Metsch and have been refined by the investigative team to enhance cultural relevance with Kazakh and Russian themes that emerge from focus groups and mapping surveys). The three components will be packaged and presented as one intervention. A more detailed description of Bridge and each intervention component is provided in the Bridge Intervention Manual.

2.2 STUDY DESIGN

Bridge will be implemented in four cities, in Kazakhstan: Almaty, Shymkent, Temirtau & Karaganda. Due to their geographical proximity of Temirtau and Karaganda, and overlapping administration of HIV clinics they will be considered as one "site" for intervention roll out. In month 16, we will use computer software to sequentially randomly assign the three sites six months prior to implementing BRIDGE in months 22, 28 and 34.

We opted for a stepped wedge trial over a cluster RCT for ethical reasons so that all clusters will eventually receive the intervention as well as to avoid contamination and to be able to control for exogenous time specific effects as discussed earlier. The unit under experimental control will be a city, with the timing of roll-out of BRIDGE in each of the study's cities—Almaty, Shymkent Karaganda, and Temirtau—to be randomly assigned. Since the cities are geographically disparate (with the exception of Karaganda and Temirtau, which is why we will consider them as a single study site) the likelihood of participant contamination is minimal.

The stepped wedge approach represents a scientific alternative to the randomized control trial (RCT) by providing a valid counterfactual to evaluate the impact of BRIDGE compared to the TP Standard of Care. This roll out design also more closely resembles the method of implementing BRIDGE in a real-world setting. The use of multiple groups and our data analytic plan will allow us to control for exogenous time-specific effects, such as introducing new HIV initiatives or funding cuts and minimizes contamination as all TP sites in a city will receive BRIDGE at the same time. More importantly, this design addresses important ethical considerations of communities by ensuring that all TP sites will eventually receive BRIDGE, which is a potentially lifesaving intervention.

The following figure describes the stepped-wedge rollout of Bridge in the four cities (with Karaganda & Temirtau combined as one study site):

Figure 2: Stepped Wedge Design

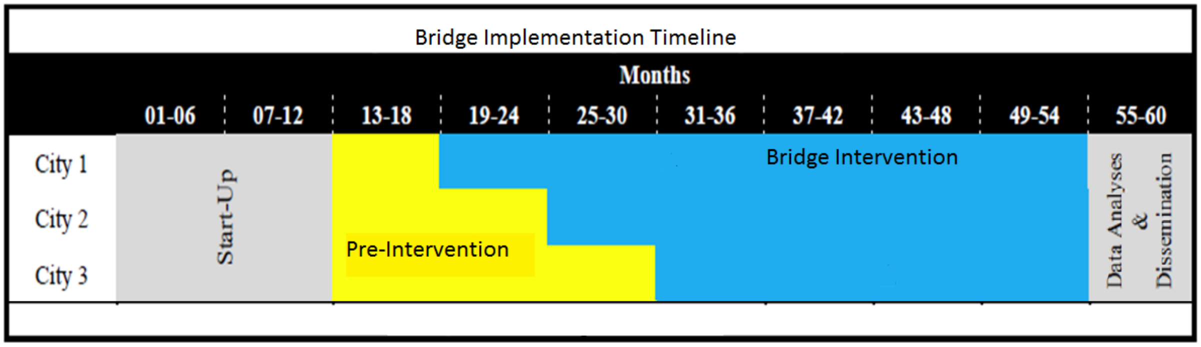

2.3. PROPOSED CONCEPTUAL FRAMEWORK FOR STUDY

The study is guided by the diffusion of innovation theory and implementation research, which identifies key multi-level factors that enhance or diminish implementation fidelity and the effectiveness of intervention delivery. We have identified the Conceptual Framework for Implementation Research (CFIR) as a guiding framework for our intervention. The CFIR framework consists of five distinct domains, each with a number of constructs serving as sub-domains (see Figure 3).

Figure 3: Conceptual Framework Guiding Implementation

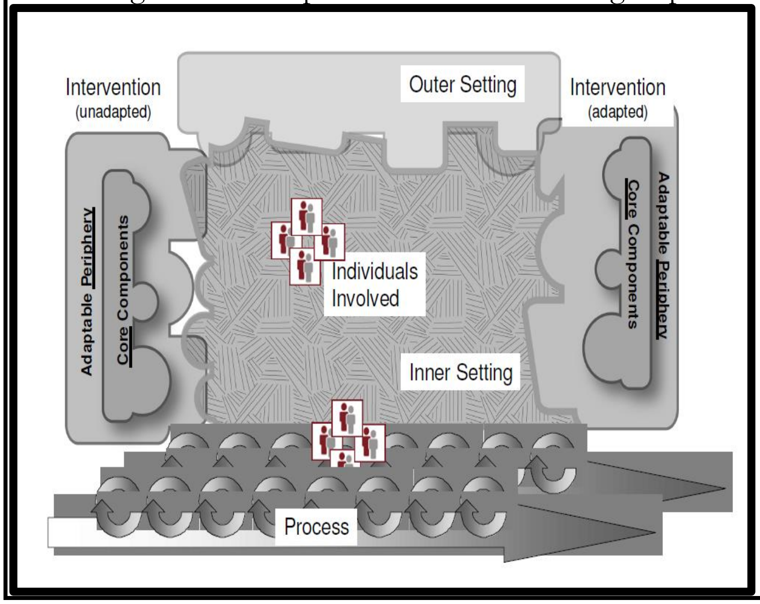

The study uses this innovative conceptual framework and employs mixed methods to identify with greater depth and precision multi-level factors (i.e. clients, staff, organization, and structural factors) which are hypothesized to influence the fidelity of implementation, effectiveness, and sustainability of BRIDGE. This mixed methods study will inform dissemination strategies to best promote the uptake and sustainability of BRIDGE in TP settings, if found to be effective.

2.4. STUDY ANALYSES

Data analyses by study aim

To test Aim 1 hypotheses, we will compare data on service linkage outcomes during post-intervention to service linkage outcomes during pre-intervention. Data for Aim 1 will be obtained from the AppSheet tracking system and process measures on ARTAS sessions.

For Aim 2, we will compare data on HIV testing in TPs, retention in HIV care, ART initiation, ART adherence, and viral suppression during post-intervention to these same data during pre-intervention. Data for Aim 2 will be obtained from the AppSheet tracking system and the AIDS Center Semi-Annual Report.

Table 1: Measurement of Study's Outcome Variables

Variable	Instrument/Measure	Time point(s)[a]	Source
Program Data			
PRIMARY OUTCOME: HIV-positive TP clients accessing HIV care	# and % of HIV-positive clients who visit HIV clinic ≥1 time in past 90 days	Ongoing (checked quarterly)	AppSheet tracking system
HIV-positive TP clients retained in HIV care	# and % of HIV-positive clients who visit HIV clinic ≥3 time in past 90 days	Ongoing (checked quarterly), semi-annually	AC semi-annual report, AppSheet tracking system
HIV testing at TP	# and % of TP clients who have received an HIV test at TP	Ongoing (checked quarterly)	AppSheet tracking system
	# of TP clients seen	Ongoing (checked quarterly)	AppSheet tracking system

<u>Statistical Analysis for Aims 1 and 2:</u> Descriptive analyses include the overall frequencies and distributions as well as estimates of parameters of interest such as measures of central tendency, dispersion, and their standard errors. These analyses also ensure proper application of the analytic procedures used to test hypotheses (e.g., distributional assumptions are met, potential outliers).

<u>Analysis of Program data:</u> Formal hypothesis testing based on program data will test for differences in outcome measures obtained during the pre-intervention period (i.e., time points before BRIDGE is implemented in a city) versus periods when BRIDGE is implemented. We will employ permutation tests (a.k.a. "randomization tests") for significance testing because of concerns that the distribution of measures may not be approximated well by a small sample size (i.e., *N*=24 TPs). In experimentally controlled trials with small sample sizes, permutation tests have been shown to be superior to parametric tests. As a heuristic and non-parametric approach, permutation testing more accurately estimates the nominal type I error rate for cluster randomized clinical trials with small sample sizes and with measures that may not be normally distributed compared to candidate parametric procedures Strong hypothesis testing in this manner involves permuting the BRIDGE implementation status of each time point; since the time period "assignment" (pre-implementation vs. BRIDGE) is allocated based on city, permutations will account for clustering by city (i.e., a permutation for TPs in the same city will all be assigned the same implementation status for that time point). Not only does this conservatively account for shared variance that might be present due to being located in the same city, statistical efficiency can be improved by clustered permutation testing. For inference, the test statistic (difference in means) observed based on actual period assignments is compared vis-à-vis the distribution of test statistics using permuted assignments, with the proportion of permuted test statistics greater than the actual observed taken as the one-sided or half of the two-sided p-value. With program measures collected quarterly over 42 months, the number of clustered permutations with 3 study sites (4 cities) is 38,416, allowing testing of statistical significance with $\alpha < .0001$. If we permute all 24 TPs independently, tests of significance can be performed with much lower α. We will conduct both permutation tests with sites permuted independently as well as clustered permutations by city and report the result with the more conservative p-value. Hypothesis testing for Aims 1 and 2—i.e., estimating the effectiveness of the intervention—will involve

examination of the population-level effect in the statistical models to make inferences about the treatment effect.

Power analyses for Aims 1 and 2: Estimates of power to detect significant differences for each hypothesis was conducted. Power analyses were conducted using G*Power (v3.1.0), two-tailed hypothesis testing, and α=.05; results are presented separately for site level data (Figure 3). For site level data, the smallest effect size for Aims 1 and 2 proportion achieving undetectable viral load; power analyses for this "rate limiting" effect size used unconditional exact test to simulate permutation testing method of estimating p-values as well as simulated cluster permutation (i.e., TPs in a city covary together). The autocorrelation and clustering effects were modeled as overall "intraclass correlation" (ICC), using a range of ICCs observed in our prior studies. Results indicate 80% power is achieved with a sample size of 24 TPs (Figure 4).

Figure 4: Estimated Power vs. Sample Size
A. Program Data (AppSheet & Semi-annual reports)

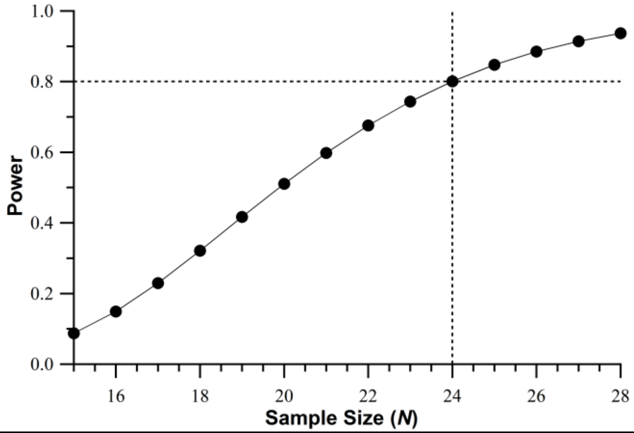

3.0 STUDY PROCEDURES

3.1. STUDY TEAM

This research will be undertaken by the Global Health Research Center of Central Asia (GHRCCA) at their regional office in Almaty, Kazakhstan. The GHRCCA was established in 2007 by Drs. El-Bassel, Gilbert and Terlikbayeva. The Center focuses on emerging global health problems such as HIV, HCV, and other infectious diseases in Central Asia. GHRCCA's main Central Asian office is in Almaty, Kazakhstan with satellite research offices in other Central Asian Republics. The Center uses rigorous social science and biomedical research to advance educational opportunities and help implement innovative public health policy solutions. This multi- disciplinary center creates crosscutting partnerships with governments, universities, business, and non- governmental organizations in Kazakhstan, the region, and worldwide to achieve its mission. The Center has built a strong infrastructure (well-trained researchers and superb media and technology systems) to allow for effective communication and shared data between the research teams in Central Asia and NYC. Ongoing training on guidelines to ensure confidentiality, security, and redundancy to avoid breaches in confidentiality and/or data loss are provided. Currently, in addition to the 22 staff, the Almaty office has a Community Collaborative Research Board and a number of faculty affiliates from Columbia University and universities in Kazakhstan, several pre- and postdoctorate students, and local and International NGO collaborators. GHRCCA has long worked with a number of local and international NGO's in Kazakhstan and has developed successful relationships with NSPs including the ones recruited for the proposed study. Since 2004, we have built solid research collaboration with Kazakhstan's Republican AIDS Center in Almaty and have established relationships with City AIDS Centers in Almaty, Karaganda, Temirtau, and Shymkent.

An organizational chart that describes the roles of GHRCCA staff and local staff is included in Appendix D.

3.2. RELATIONSHIPS WITH LOCAL PARTNERS IN EACH CITY
As an implementation science study, BRIDGE is deeply dependent on local health service organizations and

oversight bodies for both project implementation and data collection. The thorough needs assessment process during the first year of the study, which included both mapping surveys and focus groups with PWID, provided the research team with an understanding of the system of HIV care in each of the four study sites where BRIDGE will be implemented. BRIDGE staff will work with local partners in both a formal capacity (through service contracts), and an informal advisory capacity.

Service contracts:
The BRIDGE study plans to sign the following work contracts:

Shymkent City AIDS Center: guaranteeing the support of the AIDS Center administrators in all project activities, hiring staff to conduct chart review for semi-annual reports, hiring staff to complete AppSheet tracking data, hiring nursing and outreach worker staff of all 8 trust points.

Karaganda City AIDS Center: (covers project activities in both Temirtau and Karaganda, as the Karaganda AIDS Center provides oversight for the Temirtau one) guaranteeing the support of the AIDS Center administrators in all project activities, hiring staff to conduct chart review for semi-annual reports, hiring staff to complete AppSheet tracking data, hiring nursing and outreach worker staff of all 8 trust points.

Service contracts will not be signed for Almaty City due to organizational preferences.

Community Collaborative Research Board
The CCRB for the proposed study will consist of representatives from TP clients, NGOs that serve PWID, police, AIDS centers, primary care clinics and opiate substitution therapy clinics (in cities where this service is available). We will have a CCRB in each city that will meet quarterly to focus on ensuring the contextual relevance of BRIDGE and study protocols.

3.3. RECRUITMENT AND INFORMED CONSENT

<u>PWID for BRIDGE Intervention Activities</u>
As BRIDGE is an implementation study, recruitment for the BRIDGE intervention is conducted by the trust points under their normal scope of operations. Given the absence of personally identifiable data collected (the AppSheet system uses randomly assigned codes, and process measures will collect data in aggregate), we are requesting a waiver of documentation of consent for the HIV rapid testing and ARTAS components of the BRIDGE intervention, and a full waiver of informed consent for the SNS recruitment strategy. Below is a description of BRIDGE recruitment and intervention components that will be implemented in study sites over time.

The BRIDGE intervention, when it is rolled out in each study site, contains a recruitment component of peer-driven recruitment. This component is called the Social Network Strategy, and is being introduced in BRIDGE as part of a strategy for increasing the number of TP clients (see the BRIDGE Intervention Manual for more details)

We have successfully employed Social Network Strategy (SNS), a peer-driven recruitment approach, guided by network theory as a recruitment strategy in our prior studies with PWID in Central Asia. It has demonstrated effectiveness in reaching hidden and hard to reach populations of unknown HIV status and successfully linking them to HIV testing as well as re-engaging HIV-positive PWID who never initiated or were never retained in care. We clarify how we will be using peer-driven recruitment strategies below. Trust Point outreach workers will be trained to use SNS skills to teach clients to recruit 2-8 of their peers aged 18 or older. Each recruiter will receive 5-20 coded coupons and will be compensated $2 for each peer who brings a coupon to the TP and completes HIV CTR. Coupons will contain the BRIDGE logo but not CU/GHRCCA logos or an IRB approval stamp in order to emphasize to potential clients that they are receiving services from a local Trust Point and not from a research organization (more details are provided below). Peer recruiters are asked to "register" their coupon numbers with the trust point nurse, complete a short informed consent procedure (see Peer Recruiter Consent Form) and answer a brief survey. More details on the coupon management procedures and peer recruiter survey are provided in a

separate protocol, in the peer recruiter consent form, and in the peer recruiter brief survey. Peer recruiters and clients will be apprised during informed consent that the BRIDGE Program is being run at trust points as part of a research study conducted by CU and GHRCCA (and that all program materials have received approval from the CU IRB. An electronic data system designed by the project will be used to track the number of returned coupons, recruitment links, and non-client referrals using the coupon management system. Senior TP outreach workers will identify the first set of peer recruiters, through internal TP participation or referral from TP clients or other agencies. Senior TP outreach workers will train peer recruiters to identify, screen, and invite other PWID in their social networks to participate in CTR services and become peer recruiters. Senior TP outreach workers will continue to engage peer recruiters and provide ongoing coaching and support about how they can approach and effectively engage their injecting network associates using culturally tailored messages that are designed for PWID who never initiated care, dropped out of care or were not considered for ART because their CD4 count was >350. Network associates who accept CTR services will be escorted to the TP, a nurse will provide CTR at the TP and then the network associate will be engaged as a potential recruiter for SNS.

The BRIDGE intervention manual describes the SNS recruitment strategy component of the intervention, where social networks are used to bring PWID into the trust points for testing. After this initial recruitment, it is the responsibility of the trust point nurse/social worker to engage clients in rapid testing and ARTAS, as set forth in the intervention manual. All PWID who receive rapid HIV testing under the BRIDGE intervention or participate in ARTAS case management sessions will undergo an informed consent procedure to acknowledge that de-identified data on services received will be provided to the BRIDGE study. This informed consent is described in more detail below. Clients of the trust point have the right to refuse to participate in all or individual BRIDGE activities.

<u>AppSheet System</u>
Starting in November 2016, the pre-implementation phase, trust points will begin to enroll clients in a barcode-based tracking system run through the program AppSheet. This program allows the research team to collect aggregate de-identified data on what services PWID receive during their regular visits to Trust Points and the AIDS Center.

All PWID receiving services during their regular visits will undergo an informed consent procedure to acknowledge that their data will be used as part of the BRIDGE study. We are requesting a waiver of documentation of consent for this portion of the study. As trust point services are provided anonymously, a written consent form would be the only document containing the participant's identifying information.

Participants will undergo a formal informed consent process, where they will be informed that by giving consent, they are releasing their information or data about services they receive at trust points and AIDS centers as part of the research study. The informed consent form details the data that will be collected [information about the services provided to them each time they come to the trust point or AIDS Center, such as the number of syringes given, tests received (including HIV rapid tests, STI tests, viral load, and CD4 count), participation in current and/or future programming (including rapid HIV testing and the ARTAS case management sessions in the BRIDGE intervention), and the other health care facilities they were referred to, such as polyclinics, NGOs, or TB centers.], and that all data will be de-identified prior to collection. Participants will be introduced to the AppSheet system, and all data collection procedures involving this system will be described to them in detail. The consent form will also detail all potential risks and benefits to the participant in agreeing to release their data to the BRIDGE study, and all measures to protect their confidentiality will be explained in detail.

The nurse will only proceed to enroll the client into the AppSheet system if the client provides verbal consent.

The first step in enrollment into the AppSheet system is the assignment of a barcode ID (referred to as the AppSheet ID) to each PWID who comes to the trust point to receive services. The trust point nurse will explain that this is a new electronic system for keeping trust point records. He or she will explain that this AppSheet ID is a randomly assigned code that will provide them with anonymous data every time clients visit the trust point and the AIDS Center. He or she will explain that only he or she will have access to the list that links their AppSheet code

with their pre-existing trust point UIC code (see AppSheet Protocol in Appendix A for a full description of this code), and that this list will be kept securely in a locked cabinet. Clients will be encouraged to bring their ID card with the barcode printed on it to each trust point or AIDS Center visit. We encourage and will provide support for trust point nurses to create documentation for client consent to use this code. The nurse will be given talking points to explain the AppSheet program to clients, and training to prepare him or her for situations in which a client may be reluctant to use the AppSheet ID, or may refuse to use it.

When the client brings this code to an appointment, the nurse will open up the AppSheet program on a computer provided by the BRIDGE staff, and scan the client's ID code to "check in" to the trust point or AIDS Center. The AppSheet program will then ask him or her to complete a short survey about the services received that day. The surveys are provided as attachments to this IRB protocol, and they ask about the services commonly provided under trust point and AIDS Center scopes of work, as determined by the BRIDGE study team during our mapping and needs assessment procedures. Once the BRIDGE intervention begins in each study site, trust point nurses will also be asked if they carried out BRIDGE intervention activities like ARTAS counselling sessions. Nurses as well as the BRIDGE study team will be able to see the anonymous data collected through this program.

Peer Recruiter Brief Surveys will also be conducted using AppSheet. Once the peer recruiter is oriented to the SNS portion of the BRIDGE Program and has been given his or her recruitment coupons, he will be asked to visit the trust point nurse to "register" his coupon code and exchange contact information with the nurse in order to receive compensation (see more details in the BRIDGE coupon protocol). At this time, the nurse will ask if the peer recruiter would be willing to complete a brief survey with some sociodemographic and risk information. If the peer recruiter agrees, she will administer informed consent, and if consent is provided, begin the brief survey. The peer recruiter is given the option to answer the survey questions on his own, once the nurse has logged him/her in using the coupon code.

3.4. PROCEDURES FOR EACH COMPONENT OF BRIDGE STUDY

BRIDGE Intervention Procedures

As described above, BRIDGE will be rolled out in 6-month intervals between study sites (Karaganda and Temirtau will implement BRIDGE at the same time). Trust points will implement the BRIDGE intervention with guidance and support from the BRIDGE study team. The BRIDGE study team will provide initial training (described below) and follow-up trainings.

BRIDGE trust points will recruit PWID for testing through the SNS recruitment strategies, provide rapid testing for HIV in accordance with existing trust point protocols and link those with reactive tests (or known positives who have fallen out of care) to HIV care at the AIDS Centers. The intervention involves several handouts and supplementary materials as described in the BRIDGE Program Manual. In order to promote Trust Point ownership of the intervention, and to decrease any potential negative associations with CU or GHRCCA (which is affiliated with CU) we are requesting to distribute a number of supplementary materials without CU or GHRCCA logos or IRB approval stamps. These materials include recruitment materials that will be distributed throughout the community, specifically: trust point staff BRIDGE business cards, release of information form for ARTAS referrals, appointment cards for ARTAS referrals to other service providers, rapid testing informational brochure, ART informational brochure, and rapid testing coupons.
As an implementation science intervention, BRIDGE trust points will control participant consent procedures, monitoring, and retention of participants. All Trust Point staff who implement the BRIDGE intervention receive human subjects protection training from GHRCCA staff. Trust points are governed by a number of existing regulations regarding patient privacy, and all trust point services are provided anonymously in Kazakhstan. The BRIDGE intervention materials and handouts are designed to allow flexibility for trust points to adhere to their regulations: materials refer to both clients and peer recruiters by ID codes rather than names, and clients are permitted to take part in all components of the BRIDGE program anonymously, as they are permitted to receive all trust point services anonymously. The Republican AIDS Center and Almaty City AIDS Center have reviewed all

BRIDGE intervention materials to ensure that they do not violate these regulations.

The Republican AIDS Center and the Almaty City AIDS Center have approved a release of information form that Trust Point nurses can use to provide referrals to ARTAS clients during the BRIDGE intervention. This form mirrors existing release of information forms used at the Trust Points, but is adapted to BRIDGE. This release form allows for an exchange of information between the Trust Point nurse and the medical professionals at referral organizations; it does NOT allow CU or GHRCCA staff to obtain information on ARTAS client referrals, and to avoid client confusion in this regard, we plan to distribute versions of this form which do not contain the CU IRB approval stamp.

3.5. ASSESSMENTS

In addition to the activities described above, BRIDGE will use a few additional assessment tools:

<u>Semi-annual Reports from AC:</u> The research team will request de-identified treatment records of all PWID served by each city's AIDS Center, in a report format prepared by AIDS Center staff twice per year. This allows the research team to compare HIV outcomes between those clients served by a BRIDGE trust point and those served by a non-BRIDGE trust point. For a detailed description of this data collection component, please see the Semi-Annual Report Protocol in Appendix B.

We are also requesting a waiver of informed consent for the Semi-Annual Report from the AIDS Center. As described in more detail in the Semi-Annual Report protocol in Appendix B, BRIDGE study team receive this data in report format once in 6 months, with all personal identifiers removed. The staff in charge of de-identifying this data are those who already have access to it by virtue of their job positions – epidemiologists and treatment specialists at the city AIDS Centers.

3.6. BRIDGE DATA COLLECTION

A summary of all sources of data collection is provided in the table below.

Table 2: BRIDGE Data Sources

**Name**	**Description & Purpose**	**Source**	**Identification of Data**	**Pre-intervention**	**Post-intervention**
**Process measures**	Assess fidelity of BRIDGE implementation, for use in QA	Paper forms completed by BRIDGE TP nurses & OWs	Data identified by trust point, only aggregate data, no PHI or other sensitive information collected	n/a (not collected)	Forms completed, collected and reviewed monthly by site coordinators
**CFIR Surveys**	Assess implementation climate & organizational culture of BRIDGE trust points	TP, AC and RAC staff who are involved in the BRIDGE intervention	Data identified by study-assigned ID code	Every 6 months	Every 6 months
**AppSheet System**	Short surveys to assess services	Filled out by TP and AC	All PWID clients given	Continuous collection of	Continuous collection of

	received at each AC/BRIDGE TP and brief information on peer recruiters	nurses during each patient visit or at initial peer recruiter visit	random ID number (in barcode code form). All data on program level participants is de-identified and available only in aggregate form. Peer recruiters are entered into the system using the anonymous bar code on their coupons for distribution	data (pre-implementation surveys, which contain no questions about BRIDGE intervention components)	data (post-implementation surveys, which contain additional questions about BRIDGE intervention components received)
**AC Reports**	Excel-based report on biological testing & ART regimes for all HIV+ PWID in study site	Completed by epidemiological staff at AC	Data is de-identified by AC staff before GHRCCA staff are given access	Report is sent to GHRCCA in Kazakhstan once every 6 months	Once every 6 months

3.7. TRUST POINT ELIGIBILITY CRITERIA & SELECTION

Bridge will be carried out at a total of 24 trust points, or 8 per site (3 from Temirtau and 5 from Karaganda will be considered one site). A selection process within each site will identify the "BRIDGE Trust Points" who will carry out the intervention, and the "non-BRIDGE Trust Points" where no intervention will take place.

A multistage site selection process was carried out according to Figure 5:

Figure 5: BRIDGE Site Selection Process

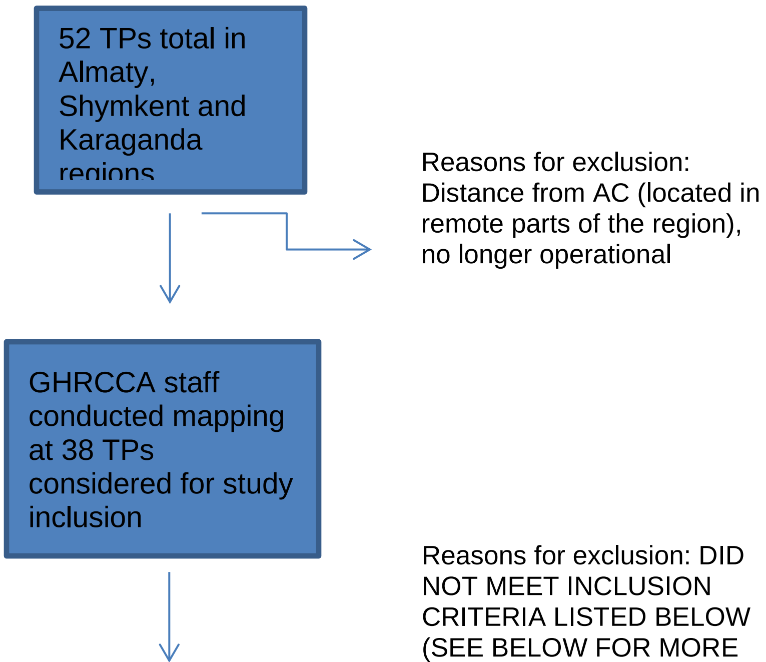

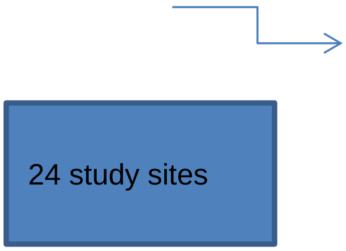

From the mapping data we collected for this project (conducted under separate IRB protocols AAAQ8761 and AAAQ6156), and by consultation by the Republican AIDS Center the following criteria were determined and will be applied in this trail:

<u>Criterion #1: Trust point must be located within 20km of AIDS Center</u>
This eligibility criterion is important given the large barrier that transportation plays in accessing care. If a trust point is located very far from an AIDS Center, it represents an additional unnecessary barrier to accessing services.

- In Almaty, 1 potential trust point was excluded for not meeting this criterion
- In Shymkent, 8 potential trust points were excluded for not meeting this criterion
- In Karaganda/Temirtau, 3 potential trust points were excluded for not meeting this criterion

<u>Criterion #2: Rapid HIV testing is an existing part of current TP services</u>
Rapid HIV testing must be currently performed in the physical location of the TP (by full-time nurse or staff or by visiting nurse/doctor). If a trust point does not currently perform rapid testing, we will not have adequate pre-intervention data to compare

In Almaty, 2 trust points do not meet this criterion because they do not currently have medical staff – NOT excluded, because they are likely to hire such staff

In Karaganda, 1 trust point does not meet this criterion

<u>Criterion #3: Physical infrastructure of TP: Separate room available for private counselling & testing</u>
Since HIV rapid testing and ARTAS activities require a private space, we have excluded all polyclinics that do not have a room for private consultation with clients. This is based on question from mapping surveys: How many rooms do you have available for private consultations with clients? TPs must report at least one to be included.

In Karaganda, 1 trust point does not meet this criterion

In Shymkent, 1 trust point does not meet this criterion

<u>Criterion #4: Trust point is located in a region which has higher numbers of injection drug users</u>

Prevalence of injection drug use is determined by GHRCCA staff in consultation with the local AIDS Center and Narcology departments. GHRCCA staff makes decisions based on neighborhood characteristics: removed trust points who don't have high prevalence of PWID in the neighborhood.

**Note that this is NOT necessarily based on the numbers of PWID that each trust point reported serving in the mapping surveys, since we are interested in potential recruits, and not a high number of PWID who are already linked to care.

**In Almaty, the trust points have been ranked in order of highest prevalence of PWID in the neighborhood, the top 8 (who meet eligibility criterion 1,2,3, and 5) are selected for inclusion.

<u>Criterion #5: Trust point staff and/or leadership are unwilling to participate in project</u>

All Trust Point sites are regulated by the RAC; therefore we have access to all the TPs and full support from the Director of RAC. However, in the event that a trust point does not wish to participate in the BRIDGE study, we cannot enforce their participation and this TP will not be included. There are many factors that may determine a trust point's willingness to participate (pressure from AIDS Center, more information about the project, staff availability), and the BRIDGE study team will be limited by this.

Of the selected 24 TP sites, about 20% will be AIDS Center TP sites operated by the Republican AIDS Center, 60% will be TP sites located and operated by public polyclinics (primary care clinics), and 20% will be independent NGO sites.

3.8. PARTICIPANTS

Categories of Participants for this study

There are three categories of participants in this implementation study:

1) PWID Program Participants: - All PWID (regardless of HIV status) in each of the four cities are considered participants in terms of program-level outcomes of BRIDGE. These clients are eligible to receive various components of the BRIDGE intervention: they may be recruited by peer recruiters as part of the SNS strategy or may be peer recruiters themselves, they will receive CTR at trust points, and those who are HIV-positive are eligible to participate in ARTAS.

Compensation (by category of participants listed above)

1) PWID program participants in the four cities receive no compensation for taking part in BRIDGE activities, as this is an implementation study. BRIDGE is designed to for trust points to provide it under their normal scope of activities. The one exception is peer recruitment: PWID who become BRIDGE peer recruiters will receive $2 compensation for every participant successfully referred to the trust point for HIV rapid testing (up to a maximum of $40), and an additional $2 for filling out a brief survey when they initially register their coupon numbers with the trust point nurse This compensation will be provided and monitored by the local project team, but distributed by the trust point nurses. No incentives are given for testing or participation in the study intervention ARTAS sessions.

3.9 QUALITY ASSURANCE

Process Measures completed during BRIDGE Implementation: To evaluate the extent and fidelity of BRIDGE intervention implementation, the study will employ a number of process and quality assurance measures. We will develop a number of monitoring forms for trust point staff (nurses and outreach workers) to complete as they perform different parts of the intervention. Local site coordinators will be responsible for monitoring this feedback, and, together with the project director, conducting regular quality assurance checks. All process and quality assurance measures are listed in the chart below:

Table 3: Study Process and Quality Assurance Measures

Variable	Measures	Time points	Source
Staff time conducting BRIDGE	Staff timesheets/logs	Weekly	TP staff (monitored by site coordinator)
Staff trained in SNS, CTR, ARTAS	# staff trainings; training attendance logs	Monthly	Project director, site coordinator
BRIDGE training quality	# training evaluations, and satisfaction surveys	Monthly	TP staff, (monitored by project director)
SNS training sessions held with peer recruiters	# coaching sessions; staff SNS log	Monthly	TP staff (monitored by site coordinator)
Quality of SNS training for peer recruiters	SNS fidelity forms	Ongoing	TP staff (monitored by site coordinator)
Network associates referred to trust point through SNS	SNS Coupon Tracking Form	Ongoing	TP staff (monitored by site coordinator)
CTR HIV Rapid Tests completed	Staff rapid testing logs & rapid testing fidelity form	Ongoing	TP staff (monitored by site coordinator)

ARTAS Enrollment & Session Completion	# ARTAS clients assigned, ARTAS sessions conducted with session log; vouchers completed	Ongoing	TP staff (monitored by site coordinator)
ARTAS Session Adherence Form	ARTAS fidelity form	Ongoing, ARTAS Sessions 1-5	TP ARTAS Staff
BRIDGE quality assurance and client coordination maintained	Attendance of TP staff at biweekly supervision meetings and number and type of technical assistance episodes	Bi-weekly	TP Nurse and Staff
BRIDGE Client Satisfaction and Peer Norms about Services	17-item client satisfaction questionnaire to assess attitudes towards TP services and BRIDGE	Ongoing	TP Clients (administered by TP staff)

4. STAFF SELECTION AND TRAINING

Each BRIDGE study site will consist of a site coordinator to oversee all site activities, 8-16 nurses/social workers implementing ARTAS and 8-16 outreach workers conducting SNS recruiting. In addition, AIDS Center staff will be trained on AppSheet Data collection. Please see the organizational chart in Appendix D for a complete description of staff structure.

General Training
All study staff will undergo a common training that covers: (1) ethical issues in research; (2) establishing and maintaining rapport with participants; (3) obtaining informed consent; (4) addressing participants' concerns with confidentiality and handling sensitive situations; (5) obtaining accurate tracking information; (6) managing distress and conducting crisis and enhanced referrals; and (7) detecting, handling, and reporting adverse events.

Training and Supervision of Data Collection Staff
Recruiters will receive an additional 25 hours of standardized training regarding data collection.

Training and Supervision of BRIDGE Intervention staff
We will provide a 7-day training for TP outreach workers, social workers and nursing staff who will implement and oversee integrated BRIDGE components in each city one month prior to BRIDGE rollout. New staff will be invited to attend subsequent trainings in other cities or make-up trainings with BRIDGE staff.. To build local capacity and further integrate the BRIDGE program into the existing TP and AIDS Center system, we have identified (with the help of Trust Point and AIDS Center leadership) a local supervisor at each study site who will take primary responsibility for all supervision, with technical and program assistance from the GHRCCA and CU study teams as necessary. This supervisor will be responsible for monthly monitoring of trust point nurses, for facilitating a monthly staff meeting (known as the community of practice) where all BRIDGE implementation staff will share their experiences and learn from each other, and by monitoring a text messaging group where these same staff can share ongoing concerns. As with all BRIDGE intervention activities, these supervision protocols have been created to align with local supervision protocols. We have developed structured manuals and implementation protocols for all BRIDGE program and supervision activities. The Study Project Director will be available to provide technical assistance to supervising nurses. Data on number and type of technical assistance contacts will be collected for Aim 4.

5. STUDY SUBJECTS

According to the Republican AIDS Center, there were an estimated total of 25,394 PWID in the 4 cities with an average of 597 registered PWID clients per TP in 2014. The 24 TP study sites should yield a pool of potential 14,328 PWID participants of whom it is estimated 13.3% on average will test positive for HIV (*n*=1,891) based on RAC surveillance reports of HIV prevalence rates in the 4 cities. This existing pool of potential TP registered clients in addition to new PWID clients recruited during the two year enrollment period should be adequate to assess

program outcomes for Aim 1 and Aim 2 outcomes.

Gender and Minority Inclusion: We anticipate that 80% of the PWID and TP staff and nurses will identify as Asian, 30% of PWID will be women, and 50% of the TP staff and HIV Care Center nurses will be women.

Participation of Children: The study will include women and men aged 18 or over, including youth 18 to 20 years of age. Younger children will be excluded because the BRIDGE intervention has not been designed for them and they are not currently served by TP. The investigative team includes certified social workers who are trained to work with young adults aged 18 to 20 years.

5.1. SCREENING AND ELIGIBILITY OF PARTICIPANTS

*E*ach category of participants is governed by its own inclusion criteria and screening procedures:

1) Program-level Participants: The BRIDGE intervention is meant to be implemented under the scope of regular trust point activities, therefore, there is no formalized screening or enrollment process. Because we will not be collecting any identifiable study data on these program level participants, we will be applying for a waiver of consent.

6. CONFIDENTIALITY OF STUDY DATA

Human subjects' protection and data safety remain an utmost priority. The primary risks are (1) psychological discomfort and/or distress arising from the personal and potentially sensitive nature of information elicited during assessments; and (2) loss of confidentiality. The level and probability of experiencing such risks are minimal, and the monitoring plan described below is commensurate with these risks. There will be ongoing monitoring of the conduct of the trial to ensure that there are no undue risks to participants, and the data are being collected in a valid and reliable way. In addition, Drs. El-Bassel, Gilbert, and the project director are directly responsible for monitoring the security of the data and the safety of participants. Approval will be received from the Columbia University IRB and the Ethics Committee of the Kazakhstan School of Public Health prior to the start of the study.

Safeguarding the confidentiality of personal data reported in screening questionnaires will be achieved via use of instruments that do not contain identifying information as defined by the Health Insurance Portability and Accountability Act (HIPAA).

RAs will verify that all screening instruments and answer sheets are gathered before they leave the data collection site. Safeguarding of program-level primary outcome data on TP clients collected through recruitment coupons and service referral vouchers will use anonymous alphanumeric codes that are not linked to any client data and will be used to generate summary statistics on overall number of TP clients recruited and number of HIV positive clients linked to HIV Care Clinics.

All hardcopy items with identifying information (e.g. brief contact information collected during screening, locator forms, biotesting release form required by the AIDS Center) will be kept in a locked file cabinet in the Project Director's office. All software files with identifying information will be stored on a computer protected by passwords and a firewall; backup copies of files will be encrypted and password protected using 128-bit AES encryption before being archived in a locked file cabinet in the Project Director's office.

<u>Data Management Systems to Preserve Confidentiality</u>

Data management activities and procedures will utilize the electronic data management systems designed by GHRCCA's investigative team to enhance the efficiency, security, and integrity of study data (including QA data), which includes: (1) using a secure and confidential World Wide Web address; (2) scheduling information for RAs, (3) automatically generating summary reports for the Project Director to tailor and Site Project Directors' participation efforts to maximize efficiency, (4) uploading of digitally-recorded and encrypted interviews for QA protocols; (4) collecting, monitoring, and summary reporting of data relevant to day-to-day operations of the study

(e.g., petty cash disbursements); and (5) using a custom-programmed data entry software to ensure consistency, integrity, and security/confidentiality of the transfer of data recorded by interviewers into a computer database. The staff will be trained to use this system by the Project Director.

Due to the need for frequent, long-distance communication, we plan to use internet-based protocols to handle international communications (e.g., Skype conference meetings, e-mails) in a cost-effective manner. We recognize that this carries inherent risks due to the number and frequency of security threats of internet-based protocols. As part of the data management training, all research staff will be informed and trained not to use any identifying information in such communications. If such information must be transmitted, it will be entered into a software file (e.g., word processing or spreadsheet document) and encrypted before electronic transmission. In addition, every effort will be made to use the more secure available method/protocol for all electronic transmissions.

We anticipate using internet-based protocols for transfer of information both regionally as well as between the study and U.S. sites. For example, file transfer protocols (FTP) will be used by study staff to transfer and transmit digital audio recordings for QA purposes. To ensure confidentiality of electronically transferred files, all files will be scrambled using 128-bit AES encryption before upload/transfer via the Internet. Files are transferred for storage on a dedicated server maintained by and accessible only to study staff. Centrally storing all software files on a password- and firewall-protected computer enhances security (e.g., all attempted accesses, whether successful or unsuccessful, are automatically logged and reviewed weekly by a dedicated SIG computer support staff member) and integrity (e.g., automatic backup of encrypted recordings onto optical media) is maintained.

At the completion of the study, all digital files with identifying information will be destroyed using software that meets DoD 5220.22-M specifications for files on magnetic media, overwriting files stored on the flash memory used by digital recorders, and physically shredded for files stored on CD/DVD media. The Project Director has extensive experience with the system and will successfully use it for this application.

These confidentiality procedures have been successfully used in prior health service research in low resource international harm reduction settings, including in other studies conducted in Kazakhstan and Central Asia. We will explain these confidentiality procedures during informed consent to potential participants.

Responsibilities

The field and analytic staff for the study, as well as the investigative staff, are responsible for immediately notifying the Project Director and PIs and completing incident reports on any breaches of protocol, breakdowns in the consent process, violations of confidentiality of the data, complaints by participants or any serious problems or adverse events. Site Project Director will be responsible for the daily management of data and for ensuring that all study staff adhere to human subjects guidelines. Oversight of data management, including data collection and storage, security, tracking, data analysis software and hardware, and QA will be the responsibility of Drs. El-Bassel, Gilbert, Terlikbayeva and the Site Project Directors who will also be responsible for ensuring that all study staff adhere to human subjects/IRB guidelines.

7. POTENTIAL RISKS

7.1. RISKS TO PARTICIPANTS FROM STUDY COMPONENTS

Risks from BRIDGE Intervention activities

The SNS, CRT and ARTAS components of the intervention may produce psychological stress. Efforts will be made to minimize this stress by assuring that well-trained and supervised TP staff will conduct BRIDGE activities. Informed consent will be waived for this portion of the study, however, before taking part in any BRIDGE activities, clients are informed that they have the right to decline to participate in any of the exercises or withdraw at any time during the intervention. Study staff will be trained in the importance of confidentiality, sign confidentiality forms, and will be certified as trained in protecting human participants in research. All TP staff delivering BRIDGE will complete a rigorous, standardized training program, and they will undergo

periodic quality assurance checks to ensure fidelity of the interventions.

Risks from HIV Testing
Social discrimination, stigma, and a loss of jobs or housing could occur if someone else learns about a participant's HIV positive rapid test results during the CTR component of Bridge. Receiving an HIV positive rapid test result may produce psychological stress. Study staff and nurses will be trained to provide standard post-test counseling when participants return for positive HIV test results.

7.2. ADDRESSING OTHER RISKS TO PARTICIPANTS

Managing Psychological Discomfort
Participants may experience anxiety or discomfort during data collection procedures. Procedures for minimizing risk to participants include the careful selection, training, and supervision of research staff together with the systematic preparation of program participants. These selection, training, and oversight protocols, which have been used in all of the prior research conducted by the investigators, have conferred an ability to answer questions that may arise. At the end of the assessment/intervention session, the research staff will ask anxious or troubled participants for permission to discuss the discomfort with the Project Director. Only with permission of the subject will the research staff discuss the subject's concerns.

Participants may become embarrassed by self-disclosed information shared during interviews. In order to reduce the risk of embarrassment and discrimination, confidentiality norms will be reviewed with research staff, and safeguards for protecting confidentiality of questionnaires and other data as outlined below will be strictly enforced. Participants at any point during the study may request that their data be destroyed without question. In addition, participants will be reminded that they have the option to skip questions and/or entire instruments.

Managing Respondent Distress
All study staff will be trained and reminded to observe verbal and non-verbal signs that may indicate that a participant is emotionally distressed. In cases where a participant is exhibiting distress, the staff member will acknowledge that the questions and activities can raise troubling issues for participants and ask if they want to take a brief break from the activity. At the end of all screening and CASI interviews, whether or not complete, RAs will ask anxious or troubled participants for permission to discuss their discomfort with the Site Project Director. Acute emergency distress referrals will be directed to the appropriate agency (e.g., hospital, mental health center) and the PI and Co-Investigators will be alerted.

Handling suicidality, homicidality, intimate partner violence (IPV) and childhood abuse:
There is no law currently in Kazakhstan that mandates researchers or clinicians to report child abuse or neglect, IPV, suicidality, or homicidality. However, if a participant indicates intent to kill or seriously injure another person or suicidal intent or if a participant indicates he or she is neglecting or abusing a child, confidentiality may not be maintained. During informed consent, participants will be apprised of these specific instances where confidentiality may not be maintained. Following protocols used in prior SIG studies with populations at elevated risk for HIV transmission, the Project Director in Kazakhstan in consultation with Dr. El-Bassel and the site coordinator will inform the appropriate agency (e.g., law enforcement or hospital). In the case of a suicidal participant, the Site Project Director will immediately accompany the participant to the hospital emergency room or a psychiatrist where he or she can be fully evaluated and will make follow-up referrals. If a participant discloses that he or she is experiencing IPV, we will assess frequency and severity of IPV, conduct safety assessment and safety planning, and provide referrals to appropriate services following a protocol that we have used other studies conducted in Kazakhstan. All incidents where staff identify participants who are involved in child abuse, IPV, or have suicidal or homicidal intent, will be reported immediately to Dr. El-Bassel and the Project Director who will be trained to assess whether these incidents may be adverse events and should be reported to the IRB. These incidents will be reviewed and discussed in the investigative team biweekly internet conference meetings.

Handling Confidentiality Issues and Cases Where There is Risk of "Imminent Harm"
Staff will be trained to report cases of child abuse or neglect and "imminent harm" to self or others to appropriate

authorities. Participants will be advised during informed consent that study staff will not be able to maintain confidentiality in cases where "imminent harm" to self or others or child abuse/neglect is suspected. If the program staff has evidence that a participant is of "imminent harm" to him/herself, the Project Director will conduct a suicidal assessment. If the Project Director assesses that the person is suicidal, s/he will notify the nurse over our study in the city where it is being conducted and that nurse will arrange for emergency psychiatric evaluation and care. If the TP staff has credible evidence that a participant is of "imminent harm" to others, the Site Project Director will notify the Medical Director and/or the local police depending on the nature and severity of the threat. Similarly, if the study staff has evidence that a participant is endangering the welfare of a child, the Site Project Director will notify the police. If a participant discloses that he or she is experiencing IPV, the Site Project Director will assess frequency and severity of IPV, conduct safety assessment and safety planning, and provide referrals to appropriate services following a protocol that we have used in other studies on HIV prevention. All incidents where staff identify participants who are suspected of child abuse or IPV or who have expressed suicidal or homicidal intent will be reported immediately to the Project Director and the PI, who will assess whether these incidents may be adverse events and should be reported to the IRBs of record and NIH. These incidents will be reviewed and discussed in the investigative team biweekly Skype internet conference meetings.

Providing Referrals for Respondents

If a TP study program staff observes signs of distress while working with clients, s/he will ask the respondent if s/he would like to discuss the issues further with appropriate professional service providers. In addition, at the end of the assessments, regardless of whether signs of distress were observed during the interview, the research assistants will ask participants how they are feeling. After allowing the participant an opportunity to express feelings raised by the interview, the interviewer will ask the respondent if s/he would like to discuss the issues further with appropriate professional service providers.

The staff member will offer the respondent a range of service options in the local community. S/he will also offer to assist the respondent in locating appropriate services and will provide enhanced referrals (e.g., calling the organization and assist in making an appointment). If the respondent is undecided about needing help and is not ready to consider a referral at the time of the interview, the interviewer will give her/him a card with the phone number and encourage the participant to contact the Site Project Director, if s/he decides that s/he would like a referral in the future. The total time spent providing referrals will be recorded as will the various types (IPV, mental health, etc.) and methods (general, enhanced) of referrals provided. Participants' responses to the referral will be assessed at all follow up measures to examine any potential impact of these referrals on primary outcomes. The investigative team has successfully used these procedures other studies.

Similar to the procedures for referrals, data regarding implementation of procedures to address suicidal/homicidal participants will be collected and analyzed in a parallel manner to investigate the effects of the research process itself on utilization/involvement with the service system and other outcomes.

Disclosure Protocol:

By virtue of the study population, it is anticipated that some of the subjects may disclose past or current engagement in lawbreaking activity (e.g., sex trading, selling drugs, theft, etc.). Following protocols used in the other studies in Kazakhstan with PWID, research staff and TP providers will inform TP clients of the limits of confidentiality at the start of session. TP staff will not release any information about a TP client and research study staff will not release information on any participants to anyone. The only exception is, as described above, when there is a possibility of "imminent harm to self or others" and/or, through group or individual discussions it becomes clear that a child is in danger or is experiencing physical abuse or neglect.

8. ADVERSE EVENTS

8.1. DEFINITION OF ADVERSE EVENTS

Serious adverse events include death, a life-threatening event, or an event resulting in hospitalization, prolongation of hospitalization, disability, and (re-)incarceration. Serious adverse events, regardless of whether or

not they are study-related, will be reported to the Columbia University IRB and the IRB at the study site serving the participant. Other adverse events include breaches of confidentiality (both intentional – e.g., mandated reporting of suicidal/homicidal participants – and unintentional), and non-life threatening psychological distress, arrest by the police and violence. Drs. El-Bassel and Gilbert will closely monitor adverse events. The investigative team is aware that the study sample is likely to experience some adverse events, though unlikely to be study-related, due to their vulnerable status in Kazakhstani society, their current or past history of law-breaking behavior (e.g. engaging in sex work) and the high levels of service needs noted earlier in this application. There is a possibility that agreements with police and service providers related to confidentiality are breached, and in this case a severe adverse event (loss of confidentiality and possible arrest of inability to obtain treatment due to arrest, for example) may occur. Drawing upon past and ongoing SIG/GHRCCA studies, the investigative team designed two forms – (1) Adverse Event: Initial Report, and (2) Adverse Event: Full Report – to document adverse events, staff responses and disposition. The procedures used to track, report, and examine adverse events are described below.

8.2. PROCEDURES FOR MANAGING ADVERSE EVENTS

<u>Protocol to Handle Each Adverse Event</u>
Study staff will identify, manage and document all adverse events. These events may be identified by RAs, the Project Director, Site Project Directors, or other project staff, including QA staff reviewing audio recordings of interviews, or they may be reported by participants. The following steps will be used to monitor, track, and document procedures used to address each adverse event and subsequent disposition:

1) Within 12 hours of an adverse event being reported/detected, the study staff member who identified the event will complete a Negative Incident Report form. The report form will include date, description of the event, duration, severity, measures taken to ameliorate the adverse event, and disposition- related information (e.g., time spent providing referrals and type of referral (domestic violence shelter or mental health treatment, etc.). The report form will be reviewed and signed by the Project Director and Dr. El-Bassel, who will be responsible for ensuring that appropriate actions have been taken.
2) Within 24 hours of the adverse event, the research staff member who identified the adverse event will discuss the event and response with the Project Director and the PI in order to ensure adherence to the protocol for handling the event.
3) Within 48 hours of the adverse event, Dr. El-Bassel, after consulting with the Project Director and any involved staff, will ascertain whether the event was related to participation in the study. A Serious Adverse Event reporting form will be completed by Dr. El-Bassel. This full report will contain an attached copy of the initial adverse event form, a summary of information obtained from other documentation, sources that clarify the nature of the event and outcomes, and the determination of whether the event was study related and by what criteria such determination was made.
4) Dr. El-Bassel and the Project Director will be responsible for reviewing the event occurrence with the appropriate staff to ensure that an adequate response is provided to the participant. This will be followed by submission of the full report to the IRBs at Columbia University and the Kazakhstan School of Public Health in Kazakhstan. In the event that a participant withdraws from the study or the investigator decides to discontinue a participant due to an adverse event, the participant will be monitored by the Project Director and the PI via ongoing status assessment until (a) a resolution is reached (i.e. a problem requiring hospitalization is stabilized with no further change expected), or (b) the event is determined to be unrelated to the study intervention.

<u>Oversight and Review of Adverse Events</u>
Every two months, the entire investigative team from Columbia University and Kazakhstan will review all adverse event data to date to determine if systematic trends exist among adverse event data to warrant changes to study protocols and procedures. Any proposed changes will be reviewed with staff at the study site, and, if needed, an external consultant (e.g., other senior investigators conducting federally-funded research on drug abuse). Approval for changes in study protocol or materials will then be obtained from the IRBs at Columbia University and from the Kazakhstan School of Public Health in Kazakhstan. After approval, the NIDA Project Scientist will be notified, and changes will also be reported in the annual progress report submitted to NIDA. The investigative team from GHRCCA at Columbia University will also conduct systematic analyses of adverse event data (e.g., associations

among frequency, type, severity of adverse events; participant characteristics; and operational aspects of research such as time-point) to inform future studies as well as recognize that such activities represent a potentially under-researched area of inquiry and scientific endeavor unto itself.

9. TRAINING AND QUALIFICATIONS OF STAFF

All research and TP staff involved will have completed the Columbia University Office of Projects and Grants' Human Subjects training course that will be translated into Russian, which has received approval from DHHS Office of Human Research Protections to supersede the NIH training course on Protection of Human Research Subjects. The Columbia University training also includes compliance with the Health Insurance Portability and Accountability Act of 1996 (HIPAA). As part of GHRCCA's standard operating protocols, all GHRCCA field, quality assurance, and management staff must complete an intensive, day-long, structured training program on detecting, addressing, and reporting adverse events before they are allowed to participate in any research.

Training which will be conducted by Drs. Terlikbayeva, El-Bassel, Hunt and Gilbert will focus on: (1) ethical issues in research, confidentiality and learning about potential adverse events that may be experienced; (2) establishing and maintaining rapport with participants; (3) obtaining informed consent from participants; (4) addressing participants' concerns with confidentiality and handling sensitive situations; (5) obtaining accurate tracking information; (6) managing distress and conducting crisis and enhanced referrals; and (7) detecting, handling, and reporting adverse events.

The training will also cover how to handle challenging situations, including how to respond to distressed participants and participants who are experiencing life threatening situations. Also, each research study at GHRCCA conducts an annual 3-hour meeting, first reviewing human subjects protection principles and study-specific procedures, followed by discussion of adverse events for the study over the past year, and role-play exercises which provide a "booster" to enable staff to handle future adverse events knowledgeably, appropriately, and expeditiously.

Qualifications and Training for Research Assistants

RAs will have a bachelor's degree and/or significant experience in conducting health and/or drug abuse research. RAs will have the capacity to conduct assessments and interview participants about sensitive topics (e.g., sexual behavior, IPV). Interviewers will receive about 40 hours of training, observation, and role-play before administering the ACASI, biological specimen collection, and pre-test counseling procedures. Interviewer training will include (1) ethical issues in research; (2) establishing and maintaining rapport with participants and different strategies of recruitment; (3) obtaining informed consent; (4) handling sensitive situations; (5) administering screening interview and determining eligibility; (6) administering ACASI and assisting participants; (7) obtaining accurate tracking information and retention strategies; and (9) managing distress and conducting crisis and enhanced referrals. The Project Director will meet weekly with RAs to reinforce adherence to study protocols and to develop consistent procedures for handling unanticipated scenarios/situations.

Qualifications, Training and Supervision of the Nurses in the TPs

For this study, nurses will be responsible for conducting rapid testing in TPs for HIV. The Project Director will participate in biweekly conference calls with Drs. El-Bassel, Gilbert, Hunt and Terlikbayeva to monitor adherence to biological testing and treatment protocols.

Timeline of Trainings for BRIDGE Staff

We will provide a 7-day training for TP outreach workers, case managers, and nursing staff who will implement and oversee integrated BRIDGE components in each city one month prior to BRIDGE rollout. New staff will be invited to attend subsequent trainings in each city. Nurses from the Trust Point will provide weekly supervision to TP staff on SNS and ARTAS activities. The local supervisor will provide bi-weekly supervision to TP nurses and conduct the community of practice monthly meetings. We developed structured manuals and implementation protocols for all BRIDGE activities. The Study Project Director with input from Dr. Hunt will be available to

provide technical assistance to supervising nurses. Data on number and type of technical assistance contacts will be collected for Aim 4.

10. BENEFITS, SUSTAINABILITY AND DISSEMINATION

10.1. BENEFITS
The main benefits to PWID of participating in BRIDGE intervention activities are having access to HIV testing and care and obtaining services, treatment, and care. The main benefits to trust point nurses, social workers and outreach workers are gaining new counselling and communication skills through the BRIDGE intervention training.

<u>Impact of Collaborative Quality Improvement Across Components:</u> Following implementation science best practices[67], we will provide continuous feedback on process measure performance to service providers in AIDS centers and TPs, rather than restrict availability of these data until the study's conclusion. We will also train case managers and AIDS center nurses on how to use these results in quality improvement across the HIV treatment continuum. We will bring a new way of delivering HIV prevention to TPs via integrating HIV care into their programs and will train the staff to use this approach in delivering the intervention.

10.3. DISSEMINATION OF FINDINGS
With completion of the BRIDGE stepped wedge trial, we will assess outcomes and contextual factors critical to successful implementation in a range of settings in Kazakhstan. To disseminate study findings we expect to write manuscripts on (1) Outcomes of study for Aims 1 and 2, (2) Description of BRIDGE and intervention (3) Implementation Facilitators and Barriers using mixed methods data from Aim 3; (4) Cost-Effectiveness of BRIDGE; and (5) Multi-level factors influencing sustainability of BRIDGE. If study results suggest BRIDGE effectiveness, this model of service integration can be used to advance HIV service delivery for PWID in Central Asia, Eastern Europe and Asia with injection-driven HIV epidemics. We will hold a scientific meeting with key stakeholders in each city to present the study findings and develop a strategic plan to scale up BRIDGE. Study findings will be disseminated through local, national, and international meetings, conferences and publications in English, Russian, and Kazakh. We will capitalize on socio-technological trends that can increase the reach and impact of study findings, such as open access publication, webinars, files and videos, social media, and metadata (e.g., search engine optimization).

<u>Appendix A: AppSheet Protocol</u>

This protocol describes all procedures and systems used in the AppSheet component of data collection for program-level outcomes for the BRIDGE study. For complete study procedures, please see the Stand-Alone Protocol.

PURPOSE OF APPSHEET DATA COLLECTION

The purpose of AppSheet data collection is to collect **de-identified** aggregate-level data on services received by PWID at BRIDGE trust points and city AIDS Centers in each of our four study sites.

The data provided in this tracking system will be used to test the hypotheses of Study Aims 1 and 2. Study Aim 1 seeks to evaluate the effectiveness of implementing BRIDGE's enhanced service integration approach in Trust Points (TPs) on linking HIV-positive People who Inject Drugs (PWID) to HIV care. Study Aim 2 seeks to evaluate the effectiveness of BRIDGE's enhanced service integration approach on increasing HIV testing in TPs and increasing retention in HIV Care Clinics. This tracking system allows us to monitor "linkage" to care and repeated testing visits among PWID by tracking what services PWID receive.

OVERVIEW OF ASSESSMENTS

AppSheet Data Collection consists of four surveys: one pre-implementation and one post-implementation survey that will be completed by trust point nurses and one pre-implementation and one post-implementation survey for AIDS Center nurses. Trust point nurses will receive compensation from BRIDGE study for collecting these data. They will receive training and supervision on use on all AppSheet Data Collection procedures by BRIDGE research staff. Prior to using AppSheet Data Collection survey, all TP nurses will complete Human Subjects Training course and submit their certification.

Pre-Implementation Survey
Prior to the start of the intervention in each city, the nurse will administer a pre-implementation survey on an electronic tablet to every client who comes to the trust point or AIDS Center. The Pre-Implementation survey will contain questions regarding which services were administered to the client. No personally identifiable information will be collected. Responses will be linked to a unique 6-digit study ID number for each client, which will consist of a code that signifies the trust point where it is created, and a random number. This ID will not be linked to the participant's name or any other PII or PHI information). The Pre-Implementation Survey will take approximately 5 minutes to complete.

Post-Implementation Survey
Following the start of the intervention in each city, the nurse will administer a post-implementation survey on an electronic tablet to every client who comes to the trust point or AIDS Center. The Post-Implementation survey will contain questions regarding which services were administered to the client, including, in the Trust Point Nurse surveys, the BRIDGE intervention components of SNS recruitment (whether the client was referred as part of the peer recruitment strategy) and ARTAS (whether the client completed an ARTAS session during that visit). No personally identifiable information will be collected. Responses will be linked to a unique study ID number for each client. The Post-Implementation Survey will take approximately 5 minutes to complete.

Study sites will switch from the pre-implementation to the post-implementation survey once BRIDGE is rolled out in their city. Due to the stepped-wedge design of BRIDGE, each study site will make this switch at a different time. A graphic is provided below, where month 13 (the start of data collection) is November 2016.

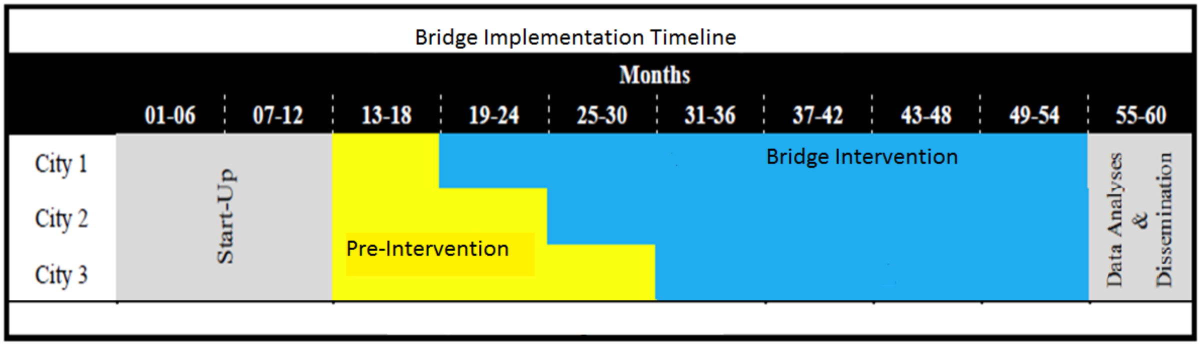

CLIENT CONSENT

Before enrolling a client into the AppSheet system, the trust point nurse must conduct informed consent with the trust point clients. As trust point services are provided anonymously, we have requested a waiver of documentation of consent.

Participants will undergo a formal informed consent process, where they will be informed that by giving consent, they are releasing their information or data about services they receive at trust points and AIDS centers as part of the research study. The informed consent form details the data that will be collected [information about the services provided to them each time they come to the trust point or AIDS Center, such as the number of syringes given, tests received (including HIV rapid tests, STI tests, viral load, and CD4 count), participation in current and/or future programming (including rapid HIV testing and the ARTAS case management sessions in the BRIDGE intervention), and the other health care facilities they were referred to, such as polyclinics, NGOs, or TB centers.],

and that all data will be de-identified prior to collection. Participants will be introduced to the AppSheet system, and all data collection procedures involving this system will be described to them in detail. The consent form will also detail all potential risks and benefits to the participant in agreeing to release their data to the BRIDGE study, and all measures to protect their confidentiality will be explained in detail.

The nurse will only proceed to enroll the client into the AppSheet system if the client provides verbal consent.

CLIENT ENROLLMENT

BRIDGE trust points are asked to enroll as many PWID clients as possible in the AppSheet system over the course of the study. "Enrollment" means that the process is explained to PWID clients, they agree to take part (as data is de-identified and void of PII or PHI identifiers, we are requesting a waiver of consent for enrolling AppSheet participants in this portion of the study (as discussed in the main protocol), and they receive an ID card with a unique ID in the form of a barcode (detailed below). In discussing the system with their clients, trust point nurses should raise the following points:

- This system is an innovative and simple way of tracking trust point clients that is being implemented at this trust point
- The system is created and monitored by a research study which is interested in how often trust point clients receive services and which services they receive
- Clients will be assigned a random ID number. The nurse is the only person who can connect this number to their UIC code (the code by which clients are registered at the trust point - see below)
- Clients should hold on to their ID card and present it every time they come to the trust point to receive needles/syringes or testing, or every time they go to the AIDS Center for any reason
- If they lose their ID card, the nurse can replace it; they will need to come back to the trust point and provide their UIC number again.

ID NUMBERS

ID numbers are assigned by the nurses at BRIDGE trust points. Starting in November 2016, trust point nurses in BRIDGE trust points will begin to assign unique 6-digit ID codes to their regular PWID clients. The 6-digit code will consist of the following components:

Digits 1-3: code of the trust point where the ID is being assigned
Digits 4-6: random digits (we suggest 001, 002, 003, etc.)

The nurse will be responsible for keeping a list of all ID codes assigned. Trust point services in Kazakhstan are anonymous, and in regular operations, nurses track clients using a standardized "unique identification code" or UIC. The nurses at BRIDGE trust points must keep a master list of BRIDGE study codes and UICs. If a client should lose their ID card (described below), the nurse can check this record and provide him with a new one based on his/her UIC.

This ID number will be converted into a scan-able bar code format and printed on a small laminated ID card that can be attached to the client's keychain.

This bar code can be scanned by a tablet or smartphone within an AppSheet survey, saving time and reducing the change of error. The keychain format is intended to prevent loss of the ID card.

DATA COLLECTION USING APPSHEET

Nurses at the trust points and AIDS Centers will collect pre- and post-implementation assessments from study participants using a secure, encrypted, password-protected tablet. No personally identifiable information or

personal health information will be collected. To minimize data entry errors and missing data, the data collection program installed on the tablet (AppSheet) will be programmed with automatic skip patterns, requirements that each item be answered or refused, and acceptance of only values within the appropriate range of responses for that item. To ensure that the correct assessment and procedures are completed at each study visit, the data collection program installed on the tablet will be programmed to include only the study assessments and procedures relevant to that visit. All data collected on the tablet will be time- and date-stamped, and uploaded to the study server.

DATA SAFETY AND SECURITY

A number of steps are taken to ensure anonymity and confidentiality of the data collected through the AppSheet System.

As trust points already provide services anonymously, through a UIC that has no relationship to any PII indicators, the assignment of an extra ID for identification within the AppSheet system represents a second layer of protection. Only the trust point nurse will have access to the list that links the AppSheet ID to their trust point UIC, and it will be stored in paper format in a locked cabinet in the trust point.

The ID cards that clients of BRIDGE TPs will be given will contain no identification information, and no indication of the participant's participation in the BRIDGE project. Should the QR code on the card be read by an outside source, the 6-digit ID would be meaningless.

All data from the data collection program installed on the tablet (AppSheet) will be collected electronically on password protected and encrypted tablets. Data and subsets of data can be limited by user, and only authorized study staff will have access to the data. Data on the tablets will be electronically transmitted through an encrypted protocol to a secure, remote server run by AppSheet. This data contains no PHI or PII only information about services received by assigned AppSheet ID number. Only authorized users will be able to log into the AppSheet server to view and download data for analysis. The server will maintain an audit trail of all log-ins, edits, and log-outs. The data will be automatically deleted from the tablet once the upload is complete and the server will send an automatic email to the US study coordinator indicating that new data have been uploaded. Access to the data collected on the tablets and stored on the AppSheet server will be limited to only those study personnel with data access rights. The US study coordinator will monitor the study server weekly to ensure that the data collected and uploaded to the server are complete and to monitor for suspicious data activity.

Appendix B: AIDS Center Semi-annual report protocol

This protocol describes the procedures for preparation of the AIDS Center Semi-Annual Report.

PURPOSE OF REPORT

The purpose of this report is to collect de-identified epidemiological and laboratory data on all HIV-positive people who inject drugs (PWID) served by an AIDS center in each of our four study sites.

The data provided in this report will be used to test the research question related to Study Aim 2, which seeks to evaluate the effectiveness of BRIDGE's enhanced service integration approach on increasing retention in HIV Care Clinic, initiating ART, and increasing adherence to HIV treatment regimens and virologic suppression. By comparing epidemiological and laboratory data between BRIDGE trust points and non-BRIDGE trust points, we will be able to evaluate the effects of implementing the BRIDGE intervention in a trust point.

DATA SOURCE

The data for the Semi-Annual Reports will be drawn from existing electronic medical records at each City AIDS Center. The Electronic HIV Case Management System (EHCMS) is a government-approved computer system for the collection, storage, transfer and analysis of epidemiological, laboratory and clinical data on all registered cases of HIV infection in Kazakhstan. Within this system, a file is created for each client who registers for treatment at the AIDS Center. All visits, tests, results and treatment information for each client are regularly entered by staff from the epidemiology and treatment department. . This system has been in place since 2011, and is used in all regions of the country. Each AIDS Center is responsible for assuring the quality of entered data through regular checks by specialists.

REPORT LOGISTICS

Regions

Data will be collected from city AIDS Centers in the four study sites: Almaty, Shymkent, Karaganda, and Temirtau.

Frequency

As this report draws data from the AIDS Centers' existing medical records, there is no need for additional data collection outside the AIDS Centers' usual scope of work. The reports will be prepared for GHRCCA every six months over the period from November 2016 to May 2020.

Staff

We will hire two staff members from each City AIDS Center to prepare these reports. These staff will be currently employed members of the AIDS Center staff who already have access to the EHCMS as part of their job:

1. Data manager - specialist from epidemiological department with access to personal data of HIV positive clients and data from national electronic tracking system for HIV cases.
2. Administrative staff member – will ensure data collection, preparation of semi-annual reports and quality assurance at the AC for GHRCCA.

DE-IDENTIFICATION OF PATIENT DATA

Data provided to GHRCCA staff will be stripped of all PII identifiers. As they are preparing the report each period, AIDS Center staff will substitute patient name with a randomly assigned BRIDGE ID (a simple four digit index number). The AIDS Center personnel responsible for report preparation will keep the only copy of the list that links the patient's name with his/her BRIDGE ID on an encrypted endpoint device. BRIDGE and GHRCCA staff will not have access to this list.

INDICATORS PROVIDED IN REPORT

Semi-annual reports shall contain following indicators:

- BRIDGE ID (as index)
- Age
- Sex
- Date when client's HIV-positive status was confirmed
- Name of the trust point where client has received services in the past 6 months
- Date registered as client of the AIDS Center
- Was the client given a CD4 test in the past 6 months?
    - Test system used for CD4
    - Results of CD4 test
- Was the client given a Viral Load test in the past 6 months?
    - Test system used for Viral Load
    - Results of Viral Load test
- Clinical Stage of Disease in the past 6 months
- Has the client begun ART?
    - Date when the client begun ART
    - Name of ART drugs (regimens)
- Changes in the client's ART regime in the past 6 months
- Changes in the specific ART medications in the past 6 months
- Testing for STIs at the AIDS Center in the past 6 months
    - STIs diagnosis in the past 6 months (if available)
    - Treatment for STIs (if available)
- Registration at the drug treatment clinic

DATA MANAGEMENT & SECURITY

<u>Data Management Systems to Preserve Confidentiality</u>

The data provided in these reports are highly sensitive, and a number of steps are taken to ensure complete confidentiality and compliance with HIPAA regulations regarding the use of medical data.

The EHCMS is a secure, computer-based system certified by the Kazakhstan Ministry of Health as a repository for sensitive health information. The system has been in use since 2011, and has the following security features: 1) access is limited only to a limited number of epidemiology department and treatment department staff; 2) the system is password protected; 3) the system is stored on encrypted endpoint devices (AIDS Center computers); 4) AIDS Center IT staff are responsible for regular security checks and protections for these devices.

Record extraction from this database is done by those who already have access to it: the epidemiological staff at each City AIDS Center.

The de-identification process, likewise, is put under the control of staff who are already authorized to access PII about each client. Each AIDS Center PWID client will be assigned a random 4-digit number (we suggest a 1001, 1002, 1003, etc. formula); the list linking client name to BRIDGE ID number will be stored in an electronic format on an AIDS Center computer. These computers are encrypted and password protected, and the epidemiologist hired to prepare the report is the only one who will have access to this list.

De-identified data will be entered into the report, which is in an Excel format. The file will be password protected. To transfer the data to the BRIDGE research team every six months, the AIDS Center will be provided with an encrypted hard drive. BRIDGE staff will receive this hard drive in-person from the AIDS Center. These study procedures ensure minimum risk of the loss of confidentiality around this data.

Data Analysis Plan for Primary Study Aim

Hypothesis:

Compared to pre-implementation time blocks, time blocks where Bridge is implemented will have increased numbers of PWID clients who (a) attend NSPs, (b) receive a rapid test for HIV at NSPs, and (c) link to HIV care at the AIDS center, when their test is positive for HIV care in the past 6 months)

Formal hypothesis testing:

Hypothesis testing draws on data from the Google app-based data collection system. The primary test for these hypotheses will be based on patient visits to NSPs, compared at the site (NSP) level. This test will utilize a repeated measures approach and will examine differences in outcome measures obtained during the time points before Bridge is implemented in a city (pre-implementation) versus time points that follow the initiation of Bridge intervention implementation (implementation) in the city. We will employ permutation tests (a.k.a. "randomization tests") for significance testing because of concerns that the distribution of measures may not be approximated well by a small sample size (e.g., N = 24 NSPs). Hypothesis testing in this manner involves permuting the Bridge implementation status of each time point; since the time period "assignment" (pre-implementation vs. Bridge intervention implementation) is allocated based on city, permutations will account for clustering by city (i.e., a permutation for NSPs in the same city will all be assigned the same implementation status for that time point). Not only does this conservatively account for shared variance that might be present due to being located in the same city, statistical efficiency can be improved by clustered permutation testing. For inference, the test statistic (difference in means) observed based on actual period assignments is compared vis-à-vis the distribution of test statistics using permuted assignments, with the proportion of permuted test

statistics greater than the actual observed taken as the one-sided or half of the two-sided p value.

This data requires an analytic approach that accommodates for the non-independence in measures arising from two sources: (1) correlations due to repeated measures with the same NSP and (2) correlations due to NSPs operating in the same city. Hypothesis testing will rely on Generalized Linear Mixed Models (GLMM) and Generalized Estimating Equations (GEE) as recommended for stepped-wedge trials. GLMM is exceedingly flexible and powerful but may not be robust to violations of normality assumptions, which will be examined using various descriptive statistics and further can exhibit difficulties in convergence in some cases. GEE is more robust; however, GEE assumes that missing data are missing completely at random while GLMM assumes that data are missing at random. The exact choice will be informed by the results from analyses that shed insight into the extent that various assumptions hold or are violated (e.g., attrition analyses, parameterization fits). In either case, sandwich estimators for variance will be used which are robust to misspecification of the correlation structure. We will use a link function that can properly model the outcome variable as a function of measurement unit or distribution (e.g., logit for binary outcomes, Poisson or Negative Binomial for count and/or rare outcomes).
