# Supplementary material for: Effectiveness of an Intervention to Improve HIV Service Delivery for People Who Inject Drugs in Kazakhstan: A Cluster Trial
Source: JAMA Netw Open. 2022 Dec 1;5(12):e2244734. doi: 10.1001/jamanetworkopen.2022.44734 (PMC9716389; doi:10.1001/jamanetworkopen.2022.44734)
Supplement: Supplement 2. — Data Sharing Statement [file jamanetwopen-e2244734-s002.pdf]

Data Sharing Statement

El-Bassel. Effectiveness of an Intervention to Improve HIV Service Delivery for People Who Inject Drugs in Kazakhstan. *JAMA Netw Open*. Published December 01, 2022. doi:10.1001/jamanetworkopen.2022.44734

Data
Data available: Yes
Data types: Deidentified participant data
How to access data: Please contact Nabila El-Bassel at ne5@columbia.edu
When available: With publication

Supporting Documents
Document types: Statistical/analytic code, Informed consent form
How to access documents: Please contact Nabila El-Bassel at ne5@columbia.edu
When available: With publication

Additional Information
Who can access the data: Data will be available for the researcher whose proposed use of the data has been approved by the PI
Types of analyses: Data will be made available for a specified purpose
Mechanisms of data availability: Data will be made available after approval of a proposal
